# Influence of Structural Determinants on Dihydrogen Adsorption and Isotopologue Separation in Nanoporous Metal–Organic Frameworks

**DOI:** 10.1021/acsami.5c24726

**Published:** 2026-02-25

**Authors:** Sibo Chetry, Prantik Sarkar, Mahnaz Bakhtian, Michael Hirscher, Harald Krautscheid

**Affiliations:** † Faculty of Chemistry, 9180 Leipzig University, Johannisallee 29, Leipzig 04103, Germany; ‡ 28325 Max Planck Institute for Intelligent Systems, Heisenbergstraße 3, Stuttgart 70569, Germany; § Institute of Separation Science and Technology, Friedrich-Alexander-Universität Erlangen-Nürnberg (FAU), Erlangen 91058, Germany; ∥ Advanced Institute for Materials Research (WPI-AIMR), Tohoku University, Aoba-Ku, Sendai 980-8577, Japan

**Keywords:** MOF, microporosity, flexible frameworks, open metal sites, hydrogen isotope separation

## Abstract

Efficient separation of dihydrogen isotopologues, particularly D_2_, is critical for applications in nuclear energy technology and environmental sciences. Conventional methods, such as cryogenic distillation, are energy-intensive and provide limited selectivity (S ≈ 1.4). Here, we report a systematic evaluation of diverse MOFs with ultramicropores, open metal sites (OMS), and framework flexibility for D_2_/H_2_ separation. Thermal desorption spectroscopy (TDS) and adsorption studies revealed that ultramicroporous MOFs enable preferential D_2_ adsorption via kinetic quantum sieving, while bimetallic Ni-MOF-74­(Co) achieves high selectivity (S = 52 at 77 K) through OMS-driven chemical affinity quantum sieving. Flexible MOFs, [Cu_2_(^n^Pr-trz-ia)_2_] and [Cu_2_(Et-trz-ia)_2_], show temperature-responsive cryogenic flexibility with selectivities of 1.4–2.3 at 77 K. These findings highlight structural design as the key to advancing dihydrogen isotopologue separation at practical temperatures.

## Graphical abstract

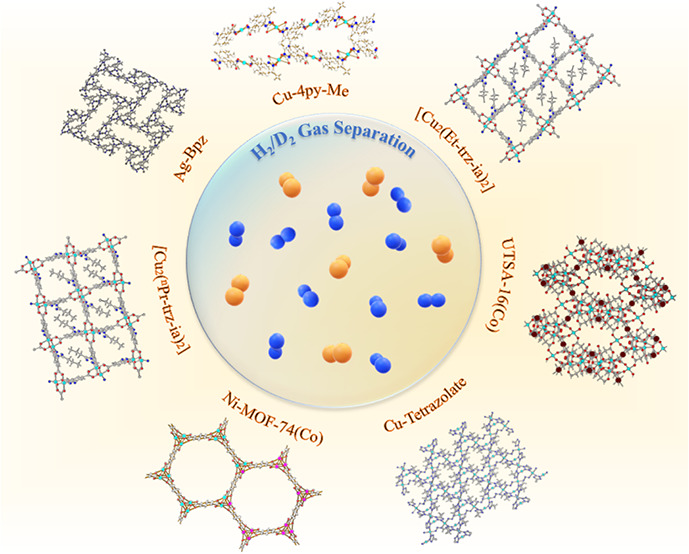

## Introduction

The heavier dihydrogen isotopologue, Deuterium (D_2_), is a critical component for a multitude of applications in both industrial and scientific fields.^1–5^ Its value is particularly evident in its usage as a primary raw material as D_2_ gas, as isotope tracer, and in nuclear reactions.^3,4^ The process of extracting D_2_ from hydrogen gas isotopologue mixtures is economically important, making it a pivotal procedure in sectors like medicinal chemistry, environmental, energy, and the field of nuclear power generation.^3,6,7^ Despite its significant demand, the presence of natural deuterium is relatively low, only 0.015% of hydrogen atoms are deuterium.^8,9^ A significant hurdle in the separation of D_2_ from its isotopologue counterparts H_2_, HD, HT and T_2_, is their remarkably similar physical and chemical characteristics, which present a substantial challenge in achieving an efficient separation using current methodologies.^9,10^ Traditional methods for the large-scale separation of hydrogen isotopes, such as the Girdler sulfide process and cryogenic distillation, though effective to an extent, are generally marked by high costs, considerable energy demands, and limited efficiency in terms of separation (separation factor S_D2/H2_ ∼ 1.4).^10^ In the quest for more efficient separation techniques, researchers have been investigating a variety of porous materials suitable for dihydrogen isotopologues separation. This research includes exploring the potential of metal–organic frameworks (MOFs), covalent organic frameworks (COFs), zeolites, and porous carbons.^2,5,11^ Among these, MOFs and COFs have shown exceptional promise, particularly in the realm of quantum sieving. They can be used to exploit both kinetic quantum sieving (KQS) and chemical affinity quantum sieving (CAQS) processes,^11,12^ demonstrating their potential in this advanced field. The KQS effect takes advantage of the differences in diffusion rates between heavier and lighter dihydrogen isotopologues in confined spaces. Heavier isotopologues have a shorter de Broglie wavelength, a smaller effective radius, and a lower diffusion barrier than lighter isotopologues, allowing them to diffuse faster in confined space.^12^ The CAQS effect is based on the isotope effect in zero-point energies, resulting in heavier isotopologues adsorbed at strong interacting sites with a higher adsorption enthalpy.^11,13,14^

Both these effects can be employed to effectively separate dihydrogen isotopologues at operating temperatures above its boiling point and have attracted immense attention. Oh et al.^15^ exploited the pore aperture to enforce the KQS effect by immobilizing functional groups (Py) onto pores of COF-1 which results in a selectivity of S­(D_2_/H_2_)= 10 at 30 K. Teufel et al. observed a selectivity of 7.5 in MFU-4 at 60 K for D_2_ over H_2_.^16^ Furthermore, the CAQS effect has been demonstrated for MFU-4l after postsynthetic treatment by exchange of Zn^II^ by Cu^I^; with a high selectivity of 11 at 100 K, making it the only reported MOF so far operating at that temperature regime.^13^ Ag^I^-exchanged NaY zeolite exhibits a D_2_ over H_2_ selectivity of S_D2/H2_ = 10 at 90 K through Ag^I^ incorporation.^17^ FitzGerald et al. first explored the CAQS effect of strong binding Ni^II^ open metal sites (OMS) in Ni-MOF-74, the selectivity reached 5 even at 77 K.^14^ Kim et al. first attempted to incorporate both the KQS effect and CAQS effect in MOF-74 by introducing a proper amount of imidazole molecules into pore channels of MOF-74 with high concentration of OMS,^18^ which achieves significantly enhanced selectivity (26) at 77 K. The presence of OMS into these materials enhanced the CAQS effect and selectivity.^13,19,20^ Enhanced selectivity was achieved using both KQS and CAQS effects in various porous materials.^13,17,21,22^

The flexibility of MOFs has been examined for purposes of dihydrogen isotopologue separation in recent studies. Selective D_2_/H_2_ separation in MOFs has been reported; examples include cobalt formate,^23^ MIL-53­(Al)^24^ and DUT-8­(Ni)^25^ which open their pores exclusively for D_2_, while blocking H_2_. Cryogenic flexibility is observed in frameworks like py@COF-1,^15,26^ and Cu-ZIF-*gis*,^27^ where the pores open at higher temperatures and remain closed at very low temperature, allowing for the adsorption and separation of dihydrogen at elevated cryogenic temperature. In addition, temperature dependent framework flexibility has been shown to play a crucial role in selective molecular sieving, as demonstrated in a zinc-based coordination network, which exhibits wide temperature separation of n-butene and iso-butene through precise aperture modulation.^28^ The KQS routes, that are based on diffusion barriers, are less effective at higher temperatures. The separation based on CAQS is limited by the rapid deactivation of OMS. Hence, the KQS method appears to be more suitable for long-term application for separation.

In this study, we synthesized a series of pristine and mixed-metal MOFs known from literature, which theoretically possess OMS and ultramicroporosity, potentially suitable for the efficient separation of dihydrogen isotopologues. These materials were chosen to reflect a diverse set of structural and functional characteristics. Mixed metal Ni-MOF-74­(Co) was selected for its well-defined open metal sites that enable strong hydrogen binding which could be further exploited for CAQS mechanism. UTSA-16­(Co) and Cu-tetrazolate were included for their ultramicroporous structure, promoting KQS-based separation. Ag-Bpz was investigated to understand the role of pore aperture at the range of 3.4 Å near to the kinetic diameter of hydrogen molecules along with high OMS density. Additionally, 1,2,4-triazolyl-isophthalate based frameworks such as [Cu_2_(^n^Pr-trz-ia)_2_] and [Cu_2_(Et-trz-ia)_2_] were chosen for their unimodal pore size matching dihydrogen’s kinetic diameter and their observed temperature-responsive gating.^29^ The choice of linker in MOF synthesis plays a critical role for the structural properties, stability, and functionality of the resulting frameworks. To explore these properties, we employed a diverse range of linkers, including a bipyrazolate,^30^ tetrazolate,^31^ cheap citrate-based linker^32^ and substituted 1,2,4-triazolyl-isophthalates.^33^ We conducted a detailed examination of the isosteric heat of adsorption (Q_ads_) and dihydrogen isotopologue separation using thermal desorption spectroscopy (TDS) and calculated D_2_ uptake and selectivity of the MOFs accordingly.

## Experimental Details

### Synthesis of MOFs

Ag-Bpz,^30^ Cu-tetrazolate,^31^ UTSA-16­(Co),^32^ [Cu_2_(Et-trz-ia)_2_], [Cu_2_(^n^Pr-trz-ia)_2_],^33^ [Cu­(Me-4py-trz-ia)][Bibr ref34] (we referred this as Cu-4Py-Me in this manuscript), and Ni-MOF-74­(Co)^35^ were synthesized and activated according to published procedures.

### Characterization and Instrumentation

Powder X-ray diffraction (XRD) patterns were obtained using a STOE STADI-P diffractometer with Cu–K_α1_ radiation (λ = 1.54060 Å). The samples for these measurements were prepared in glass capillaries (Hilgenberg, outer diameters 0.5 mm or 0.7 mm). SEM images were captured using a Phenom Pharos G2 Desktop FEG-SEM Tabletop field emission gun scanning electron microscope for high quality imaging (acceleration voltage 15 kV, back scattered electron detector). The nickel and cobalt concentrations were also measured quantitatively using ICP-OES on a PerkinElmer Optima 8000 instrument. For sample preparation, the MOFs were digested in nitric acid. X-ray photoelectron spectroscopy (XPS) analysis was conducted using a K Alpha+ XPS system (Thermo Fisher Scientific Instruments, UK). For the measurements, monochromatic Al–Kα radiation was utilized, generated in a sealed X-ray tube with a beam current of 6 mA and an acceleration voltage of 12 kV. Instrument calibration was verified using the Ag 3d peak at 352 eV. The spot size for analysis on the sample was 400 μm. Binding energies were referenced to the C 1s peak at 284.8 eV.

### Gas Adsorption

Adsorption isotherms up to 100 kPa were measured using a BELSORP MAX and BELSORP MAX G (Microtrac MRB) equipped with a 3P cryoTune for temperatures higher than 77 K. Before the measurement, all the samples were activated according to their reported activation procedure. The measurements were conducted in the pressure range of 10^–2^ – 100 kPa. All the gas adsorption measurements were performed using gases of high purity (H_2_: 99.98% and D_2_: 99.98%). Pure Helium (purity: 99.998%) was used in all cases to measure the dead volume.

### Dihydrogen Isotopologue Separation

Isotope separation performance was investigated using thermal desorption spectroscopy (TDS). In this technique, the sample was first introduced to a 1:1 isotopic mixture of D_2_ and H_2_ at various exposure pressures *p*_exp_ (10–100 mbar) and exposure temperatures *T*_exp_ (30–77 K). Following this step, the remaining nonadsorbed gas molecules were pumped off using a turbomolecular pump (∼10^–7^ mbar). Then the sample was further cooled down to 20 K to preserve the adsorbed state. The final step involved applying a linear heating ramp (0.1 K s^–1^) from 20 to 300 K. A quadrupole mass spectrometer was used to continuously monitor the desorbing gas, detecting a rise in pressure in the sample chamber as the gas desorbed. The instrument was calibrated by using Pd_95_Ce_5_ metal alloy. The selectivity for D_2_ relative to H_2_ (S_D2/H2_) was calculated by integrating the area under the desorption peaks, which is proportional to the desorbed amount of each species.

## Results and Discussion

### Characterization of MOFs

All MOFs were synthesized following procedures described above, and their crystallinity and phase purity were confirmed by PXRD. The obtained diffraction patterns match well with the corresponding simulated patterns (SI S4, Figure S8). In addition, Pawley refinement further supports the phase purity of the materials. These findings highlight that the synthetic approach employed here reliably yields phase-pure MOF materials, suitable for further investigations of their structural and functional properties. Mixed-metal Ni-MOF-74­(Co) was characterized by SEM-EDAX and ICP-OES, providing evidence that Ni was successfully incorporated into MOF-74­(Co) with a 1:1 Ni:Co ratio (SI S5, Figure S9).

Furthermore, all the MOFs were analyzed using Zeo++,^36^ a tool package made for the investigation of crystalline porous materials, complementing the findings. It provides both single-crystal data and high-throughput analysis of huge databases, and it enables geometry-based evaluation of empty space, structural topology, and alterations or assembly of structures. This analysis demonstrates that most of them have ultramicropores (pore diameter <7 Å). MOFs like UTSA-16 (Co), Cu-4Py-Me, and Cu-tetrazolate only have ultramicropores, and Ag-Bpz exhibits a pore aperture of 3.5 Å with major contributions from supermicropores (3.5 and 8 Å), whereas Ni-MOF-74­(Co) has micropores wider than 10 Å (Table 1). Zeo++ analysis also identified open-metal sites (OMS) in Ag-Bpz and Ni-MOF-74­(Co). Ag-Bpz and MOF-74­(Co) exhibit OMS densities of 7.9 and 7.5 mmol cm^–3^.

**Table 1:** Comparison of Pore Size, OMS Density, Gas Uptake and Heat of Adsorption of Several Porous Materials

MOFs	Pore size (Å)	Open metal sites density (mmol cm^^–3^^)	Hydrogen uptake at 77 K and 100 kPa (mmol g^^–1^^)	Isosteric heat of adsorption Q_ads_ (kJ mol^^–1^^) at low coverage
Ag-Bpz	3.5–8	7.9	3.7 (H_2_)	7.0 (H_2_)
			4.5 (D_2_)	8.0 (D_2_)
Cu-4Py-Me	5.5	-	15.6 (H_2_)	6.5 (H_2_)
			16.5 (D_2_)	7.3 (D_2_)
Cu-tetrazolate	3.2–3.6	-	0.7 (H_2_)	9.6 (H_2_)
			1.2 (D_2_)	11.1 (D_2_)
UTSA-16(Co)	3.4–4.4	-	3.7 (H_2_)	8 (H_2_)
			4.4 (D_2_)	10 (D_2_)
[Cu_2_(Et-trz-ia)_2_]	3.0–3.4	-	4.9 (H_2_)	12.0 (H_2_)
			5.1 (D_2_)	13.4 (D_2_)
[Cu_2_(^^n^^Pr-trz-ia)_2_]	3.0–3.4	-	5.9 (H_2_)	12.3 (H_2_)
			6.1 (D_2_)	14.2 (D_2_)
Ni-MOF-74(Co)	11	7.5	7.4 (H_2_)	11.6 (H_2_)
			8.2 (D_2_)	12.6 (D_2_)

### Gas Adsorption Properties

Furthermore, to investigate the adsorption behavior of the MOFs toward dihydrogen isotopologues, H_2_ and D_2_ adsorption isotherms were recorded at 77 and 97 K. From these measurements, the isosteric heat of adsorption (Q_ads_) was determined using the Clausius–Clapeyron equation.^37^ The calculated values are characteristic for MOF-74-type materials, reflecting the presence of OMS. At low coverage, Ni-MOF-74­(Co) with OMS exhibits high values of Q_ads_, reaching 12.6 kJ mol^–1^ for D_2_ and 11.6 kJ mol^–1^ for H_2_ (SI S7, Figure S17). These values are slightly higher than those reported for pristine MOF-74­(Co) with 11 kJ mol^–1^ for H_2_ and 12.1 kJ mol^–1^ for D_2_,^20^ indicating that Ni incorporation enhances the interaction between H_2_ molecules and the metal centers. This strengthening can be attributed to the higher Lewis acidity of Ni^2+^ relative to Co^2+^, which leads to shorter H_2_···metal distances and a larger induced dipole in the adsorbed hydrogen molecules. In contrast, Ag-Bpz, despite possessing a high density of OMS ∼7.9 mmol cm^–3^, (SI S3.1, Table S1), shows much lower heats of adsorption (8.0 kJ mol^–1^ for D_2_ and 7.0 kJ mol^–1^ for H_2_). The hindered binding strength could originate from the 4-fold interpenetrated (10,3)-a coordination network structure,^30^ which restricts accessibility to the metal sites and creates a coordination environment less favorable for strong dihydrogen binding compared to Ni-MOF-74­(Co).

Similarly, UTSA-16­(Co), [Cu_2_(^n^Pr-trz-ia)_2_], [Cu_2_(Et-trz-ia)_2_], and Cu-tetrazolate show Q_ads_ around 10–14.5 kJ mol^–1^ for D_2_. Therefore, in materials with smaller pore sizes within 3–7 Å (SI S7, Figure S26 a, b), the confined space allows van der Waals potentials from neighboring atoms to overlap, potentially generating regions of pronounced surface curvature and thereby leading to unusually strong interaction energies between the pore walls and the adsorbed gas molecules.^38–41^ Also, it is observed that due to the quantum statistical mass effect, the D_2_ uptake is always somewhat greater than that of H_2_ under the same conditions (Table 1 and SI S7, Figure S26a).^42^ This difference is reflected in the isotherms, where D_2_ adsorption is slightly higher relative to H_2_, particularly at low pressures. The corresponding heats of adsorption Q_ads_ follow the same trend, with D_2_ binding more strongly than H_2_. A clear correlation emerges between pore size and Q_ads_ for frameworks with pore dimensions close to the kinetic diameter of H_2_, they display enhanced heats of adsorption, while an increase in pore size generally leads to weaker interactions. An exception to this trend occurs in MOFs with OMS, such as Ni-MOF-74­(Co), where the strong metal–adsorbate interaction dominates over simple pore confinement effects. In contrast, Cu-4Py-Me does not exhibit accessible OMS, and its adsorption behavior is instead governed primarily by the pore environment, resulting in comparatively lower Q_ads_ values despite its porosity. It shows a moderate and uniform heat of adsorption (7.3 kJ mol^–1^ for D_2_ and 6.5 kJ mol^–1^ for H_2_). In Cu-4Py-Me a uniform pore environment due to polar functional groups leads to adsorption sites having similar energies which is a consequence of the low partial positive charges of the copper atoms as determined by electronic structure calculations.^43^ Out of the investigated samples, Cu-4Py-Me shows the highest D_2_ uptake of 16.5 mmol g^–1^ at 77 K and 100 kPa, followed by 8.4 mmol g^–1^ for Ni-MOF-74­(Co). D_2_ uptake values of the other materials were in the range of 2–5 mmol g^–1^, with Cu-tetrazolate showing the lowest uptake of 1.2 mmol g^–1^ (Table 1).

### Dihydrogen Isotopologues Separation

To investigate the separation efficiency of these MOFs, thermal desorption spectroscopy (TDS) experiments were performed. The underlying mechanism for D_2_/H_2_ separation using ultramicroporous MOFs is Kinetic Quantum Sieving (KQS). KQS arises from the difference in thermal de Broglie wavelength of H_2_ and D_2_, which leads to the distinct effective diffusion barriers within the narrow pores. Since the thermal de Broglie wavelength (λ) is inversely proportional to the square root of temperature, the magnitude of KQS effect substantially increases at sufficiently low operating temperature. Accordingly, an exposure temperature of 30 K was selected as a standard condition for comparative evaluation of all the MOFs investigated in this study. Measurements at lower temperatures (20 and 25 K) were deliberately excluded because partial condensation of D_2_ under these conditions can give rise to additional small peaks or enhanced signals in the TDS spectra, potentially distorting the calculated D_2_/H_2_ selectivity and obscuring fundamental conclusions of this study. In cryogenic TDS experiments on MOFs, exposure pressures in the range of 10–300 mbar have been previously used to achieve sufficient gas uptake without saturating all the adsorption sites. At low pressure (<10 mbar), adsorption is largely confined to the strongest binding sites, whereas at significantly higher pressures (>1 bar), multilayer adsorption and pore filling can mask intrinsic quantum sieving effects. Therefore, *p*_exp_ = 100 mbar was selected as a standard pressure to ensure measurable adsorption while keeping intrinsic D_2_/H_2_ selectivity intact. An exposure time of *t*_exp_ = 10 min was also adopted as a standard parameter, consistent with previous cryogenic isotope separation studies. For example, Ha et al., have measured a series of Hofmann-type MOFs with high OMS density at *t*_exp_ = 10 min to quantify desorption and isotope selectivity.^19^ Shorter exposure time limits diffusion into the pore network, thereby emphasizing kinetically accessible adsorption sites and minimizing diffusion related artifacts, which is important when comparing ultramicroporous, flexible and OMS containing MOFs.

All the ultramicroporous MOFs were exposed to a 1:1 H_2_:D_2_ mixture with *p*_exp_ = 100 mbar at a temperature of *T*_exp_ = 30 K, the resulting TDS spectra are presented in Figure 1. In a typical TDS spectrum, weak adsorption sites show up as increasing desorption rates at low temperature, followed by stronger adsorption sites with desorption at elevated cryogenic temperature. Major desorption maxima of Cu-4Py-Me are positioned around 56 K for D_2_ and 50 K for H_2_ (Figure 1a) after exposure to an equimolar D_2_-H_2_ mixture. The presence of a small bump at a relatively low temperature (35 K) indicates another weak binding site as well. This MOF exhibits moderate selectivity S_D2/H2_ = 8, with a high D_2_ uptake of 9.7 mmol g^–1^. Under the same experimental conditions, UTSA-16­(Co) demonstrated desorption peaks at 53 K for D_2_ and 41 K for H_2_ (Figure 1b) and, in addition, there is a shoulder at 40 K in the TDS of D_2_, suggesting the presence of two different adsorption sites. UTSA-16­(Co) illustrates the trade-off between selectivity and D_2_ uptake, as it shows higher selectivity (S_D2/H2_ = 15) but compromised D_2_ uptake (2.7 mmol g^–1^) compared to Cu-4Py-Me. Similarly, Cu-tetrazolate at *T*_exp_ = 30 K displays a maximum desorption temperature of 41 K for D_2_ and 40 K for H_2_ (Figure 1c). While the selectivity is highest among all the investigated ultramicroporous MOFs, however, its D_2_ uptake is much lower, only 0.2 mmol g^–1^, due to its limited porosity. Furthermore, Ag-Bpz exhibits two desorption peaks for both gases (Figure 1d), with moderate selectivity of S_D2/H2_ = 5.5 and D_2_ uptakes of 3.8 mmol g^–1^, demonstrating that the highly interpenetrated structure of Ag-Bpz leads to lower selectivity and uptake in contrast to other MOFs.

**Figure 1:**
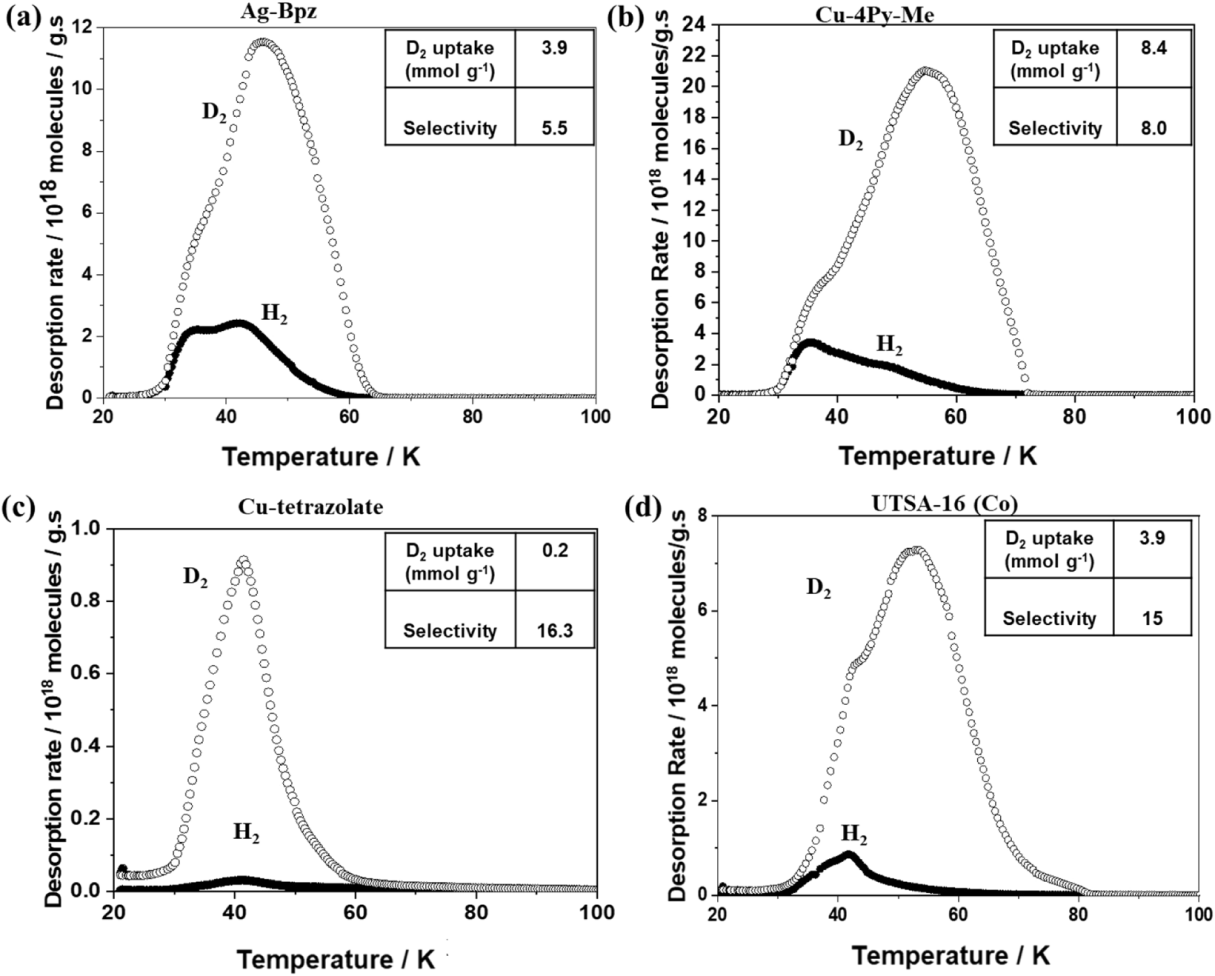
Thermal desorption spectroscopy (TDS) graphs of (a) Ag-Bpz, (b) Cu-4Py-Me, (c) Cu-tetrazolate, (d) UTSA-16­(Co) at exposure temperature *T*_exp_ = 30 K. The samples are exposed to an equimolar mixture of H_2_ and D_2_ at exposure pressure *p*_exp_ = 100 mbar for 10 min. H_2_ and D_2_ are represented by closed and open circle symbols, respectively.

TDS spectra of Ni-MOF-74 (Co) at different exposure temperatures (30 and 77 K) are presented in Figure 2. For *T*_exp_ = 30 K, two distinct broad desorption peaks are recorded for D_2_ (47 and 83 K) and one for H_2_ (35 K). The slight shift of the first desorption maximum to higher temperature for D_2_ is attributed to the difference in binding energy between gas molecules (H_2_ vs D_2_) and the MOF surface, as explained by Oh et al.^20^ The desorption maximum below 60 K is assigned to the benzene rings and triangular oxygen sites in the MOF-74 framework.^20^ On the other hand, the second desorption maximum arising above 70 K is attributed to the D_2_ molecules released from OMS.

**Figure 2:**
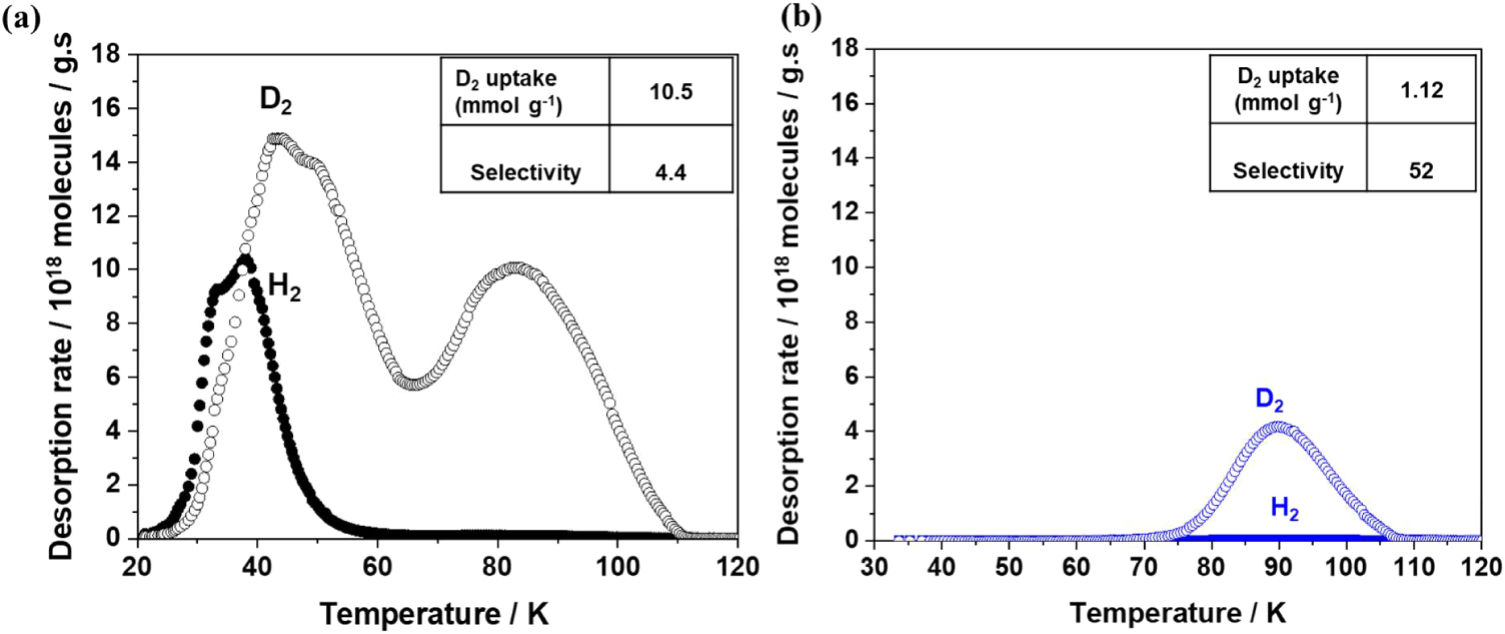
Thermal desorption spectroscopy (TDS) graphs of mixed-metal Ni-MOF-74­(Co) at exposure temperatures (a) *T*_exp_ = 30 K, (b) *T*_exp_ = 77 K. The samples are exposed to an equimolar mixture of H_2_ and D_2_ at exposure pressure *p*_exp_ = 10 mbar for 10 min. H_2_ and D_2_ are represented by closed and open circle symbols, respectively.

These observations reveal the existence of strong adsorption sites that are typical of MOF-74-type frameworks with OMS. The D_2_ uptake of 10.6 mmol g^–1^ is also similar to the previously reported data for the MOF-74 family. However, at higher exposure temperatures (77 K), Ni-MOF-74­(Co) shows a single desorption peak with D_2_ uptake of 1.2 mmol g^–1^ and a high D_2_/H_2_ selectivity of S = 52, due to the contribution only from the strong adsorption sites. In comparison to the study by Oh et al.^20^ on pure MOF-74­(Co) at *T*_exp_ = 30 K, the selectivity (∼4) and D_2_ uptake (∼10 mmol g^–1^) of Ni-MOF-74­(Co) are very similar. In contrast, at *T*_exp_ = 80 K, the selectivity of MOF-74­(Co) increases to only 6.3. Surprisingly, Ni-MOF-74­(Co) demonstrates a selectivity two times higher than MOF-74­(Ni)^18^ and about nine times higher than pure MOF-74­(Co),^20^ indicating the strong influence of Ni incorporation on dihydrogen isotopologue separation. Higher partial positive charge of Ni^2+^ ions (1.50 vs 1.42 e^–^ for Co^2+^) amplifies polarization interactions, leading to enhanced contributions from both permanent electrostatics and induced polarization. Ni-MOF-74 induces greater dipole (0.25–0.55 D) on the adsorbed H_2_ molecules compared to Co-MOF-74 (0.22–0.50 D), resulting in a stronger Ni^2+^-H_2_ interaction than Co^2+^-H_2_.^44^

In case of Ni-MOF-74, the calculated distance between Ni^2+^ and the center of mass of the adsorbed H_2_ molecule is about 2.34 Å, which is in fact the shortest within the M-MOF-74 series (sequence of M-H_2_ distance: Ni-MOF-74 < Co-MOF-74 < Mg-MOF-74 < Zn-MOF-74). The calculated Co^2+^···H_2_ distance of 2.45 Å is in agreement with neutron powder diffraction studies.^14,44^ Therefore, the Ni^2+^ center was deliberately chosen to be introduced into Co-MOF-74. Thus, the introduction of Ni^2+^ centers in Co-MOF-74 enhances the zero-point energy difference between H_2_ and D_2_ relative to pristine Co-MOF-74. In contrast to KQS, CAQS is governed by the strength of interaction between dihydrogen isotopologue molecules and strong adsorption sites, resulting in much higher selectivity of Ni-MOF-74 (Co).

[Cu_2_(^n^Pr-trz-ia)_2_] and [Cu_2_(Et-trz-ia)_2_] exhibit framework flexibility, which was confirmed by adsorption isotherms recorded at 195 K (SI S7, Figure S25). To understand the role of framework flexibility in these paddle wheel MOFs, an additional experimental procedure was applied using the TDS technique alongside the regular method: An isotopologue mixture was first exposed to the MOFs at *p*_exp_ = 100 mbar and *T*_exp_ = 77 K to enable pore access, followed by evacuation at 30 K. For [Cu_2_(^n^Pr-trz-ia)_2_], exposure at 30 K resulted in a main desorption maximum at 65 K, with a small shoulder near 38 K, a D_2_ uptake of 0.1 mmol g^–1^, and a selectivity of 4.6 (Figure 3). When exposed at 77 K, the same material displayed a single desorption peak at 80 K, a notably higher D_2_ uptake of 1.95 mmol g^–1^, and a reduced selectivity, consistent with increased pore accessibility at higher temperatures. A similar trend was observed for [Cu_2_(Et-trz-ia)_2_], which showed desorption maxima at 39 K (D_2_) and 37 K (H_2_) at *T*_exp_ = 30 K with negligible D_2_ uptake (0.02 mmol g^–1^) but high selectivity of S_D2/H2_ = 5.6. At *T*_exp_ = 77 K, the uptake rose to 0.8 mmol g^–1^, with a single desorption peak at 90 K for D_2_ and 89 K for H_2_, and selectivity decreased to 2.3. In both MOFs, D_2_ desorbed at slightly higher temperatures than H_2_, indicating stronger binding interactions. Although the selectivity declined at 77 K compared to 30 K, the overall performance of these flexible MOFs remains superior to commercial cryogenic distillation (S_D2/H2_ = 1.4 at 24 K), highlighting their potential as advanced materials for dihydrogen isotopologue separation at more practical operating temperatures.

**Figure 3:**
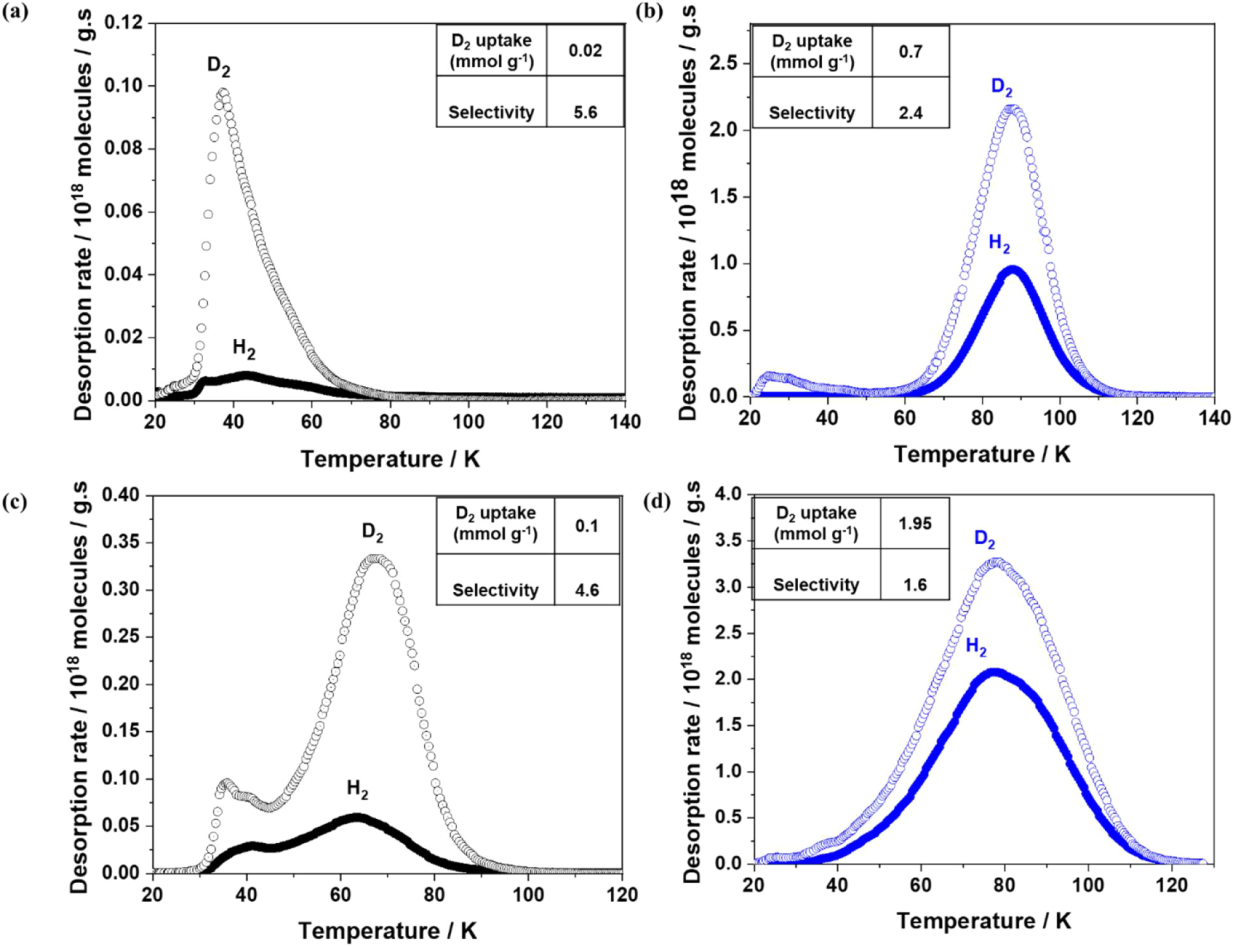
TDS spectra of [Cu_2_(Et-trz-ia)_2_] at (a) *T*_exp_ = 30 K and (b) *T*_exp_ = 77 K; TDS spectra of [Cu_2_(^n^Pr-trz-ia)_2_] at (c) *T*_exp_ = 30 K and (d) *T*_exp_ = 77 K. The samples are exposed to an equimolar mixture of H_2_ and D_2_ at exposure pressure *p*_exp_ = 100 mbar for 10 min. H_2_ and D_2_ are represented by closed and open circle symbols, respectively.

Ultramicroporous MOFs (Cu-tetrazolate, UTSA-16, Cu-4Py-Me, Ag-Bpz), lacking structural flexibility and accessible OMS display a direct correlation between pore aperture and isotopologue selectivity. When the entrance pore diameter approaches the size of a gas molecule, which is in the same magnitude as the molecule’s de Broglie wavelength, this condition increases the diffusion barrier for lighter isotopologues due to quantum effects. At low temperatures, heavier isotopologue molecules therefore diffuse faster than lighter ones within porous materials. This behavior leads to KQS driven separation. Consequently, as the pore size increases from 3.4 Å (e.g., Cu-tetrazolate) to 5.5 Å (e.g., Cu-4Py-Me), a pronounced decrease in the D_2_/H_2_ selectivity is observed (Figure 4). In contrast, flexible MOFs exhibit reduced selectivity with increasing temperature, attributable to thermally induced framework dynamics that diminish sieving effects. Notably, in the case of Ni-MOF-74­(Co), high selectivity is maintained despite its relatively large pore size (∼10 Å), owing to the presence of OMS that facilitate strong, selective interactions with D_2_ even at cryogenic temperatures (77 K). All MOFs studied here are compared to well-known other examples summarized in Table S4 (SI S8), which provides a direct benchmark for their adsorption and separation performance.

**Figure 4:**
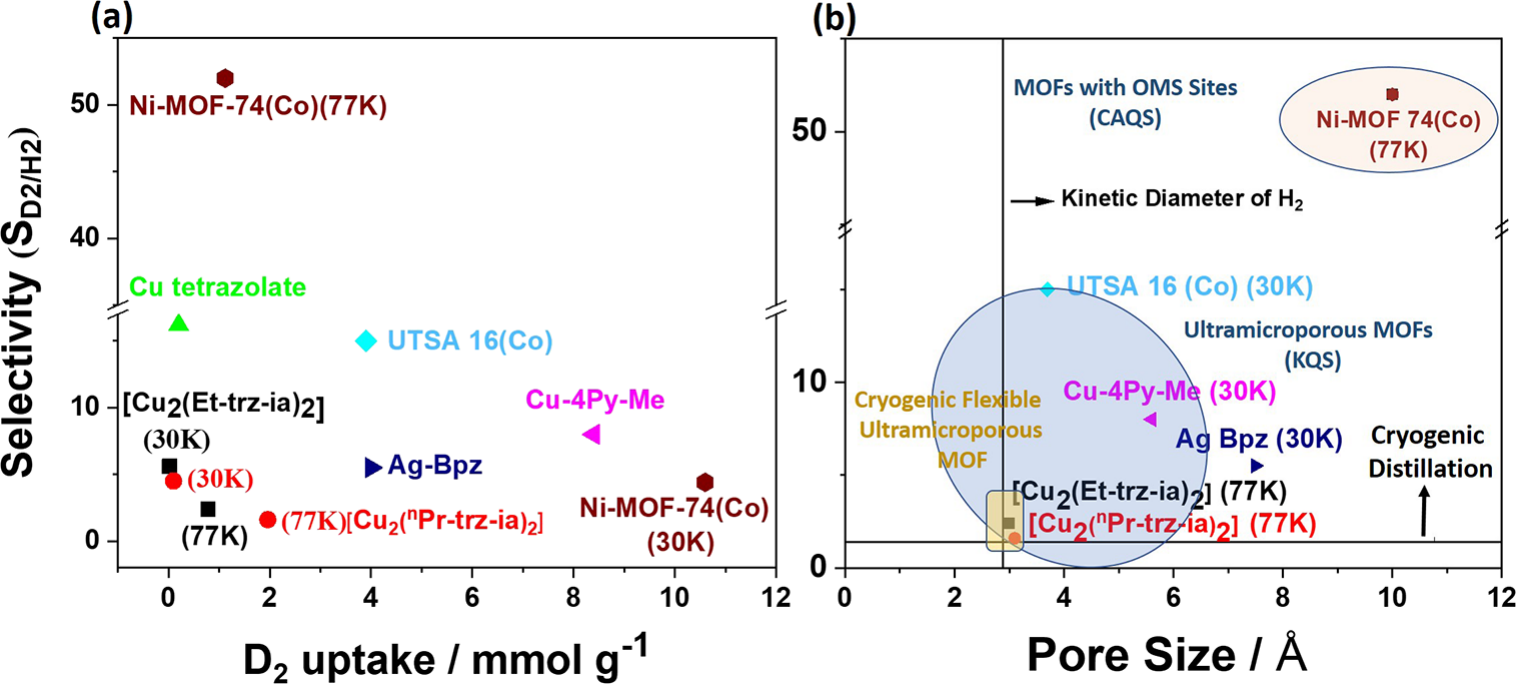
(a) Selectivity plotted as a function of corresponding D_2_ uptake (mmol g^–1^), (b) D_2_/H_2_ selectivity calculated from TDS experiment plotted as a function of pore size (Å) of the MOFs. The horizontal and vertical black lines symbolize the selectivity of the cryogenic distillation process and the kinetic diameter of H_2_ molecules, respectively.

Moreover, selectivities calculated from pure gas adsorption isotherms were also estimated using IAST calculations using the robust python-based package pyGAPS (Python General Adsorption Processing Suite).^45^ For ultramicroporous MOFs, the calculated selectivities (SI S10, Table S5) are in good agreement with the TDS-derived results, demonstrating the reliability of IAST in systems without OMS. While Kim et al.^18^ have already reported that for MOF-74­(Ni), the IAST-derived selectivities deviate significantly from TDS measurements, a similar discrepancy is observed here for Ni-MOF-74­(Co). This highlights that in frameworks containing OMS, the specific strong interactions between H_2_ or D_2_ and OMS are not suitably modeled by IAST, leading to misleading selectivity values. Therefore, while IAST provides reasonable predictions for ultramicroporous MOFs without OMS, it should be applied with caution or avoided for MOFs with accessible metal centers when evaluating dihydrogen isotopologue separation performance.

## Conclusions

This systematic study identifies how pore size, open metal sites (OMS), linker properties, and cryogenic flexibility govern D_2_/H_2_ separation in MOFs, establishing design principles applicable to future materials development.

Three distinct mechanistic pathways emerge from comparative analysis. Ultramicroporous frameworks operating in the 3.0–4.5 Å regime achieve kinetic quantum sieving (KQS) through de Broglie wavelength differences. Cu-tetrazolate (3.2–3.6 Å, S_D2/H2_ > 16 at 30 K) and UTSA-16­(Co) (3.4–4.4 Å, S = 15) demonstrate high selectivity, although with inherently limited uptake. Cu-4Py-Me (5.5 Å, S_D2/H2_ = 8) represents a practical compromise, delivering 10.9 mmol g^–1^ uptake with moderate selectivity. The mechanistic basis reflects how pore confinement creates differential diffusion barriers: D_2_, possessing a shorter de Broglie wavelength and lower zero-point energy, navigates confined spaces more readily than H_2_. Linker selection controls this selectivity-capacity trade-off. In Cu-tetrazolate and citrate-based MOFs, the rigid linkers generate the tightest pores optimal for KQS.

OMS drive a complementary mechanism: chemical affinity quantum sieving (CAQS). Ni-MOF-74­(Co) achieves S_D2/H2_ = 52 at 77 K via high OMS density (∼7.5 mmol cm^–3^) and strong metal–adsorbate interactions (Q_ads_ > 12 kJ mol^–1^ for D_2_). Metal centers prove decisive: Ni^2+^ with its higher partial charge outperforms Co^2+^ by a factor of 9, attributed to higher Lewis acidity, induced dipole formation, and shorter M···H_2_ contact distances. An advantage of CAQS is its maintained high selectivity even at elevated cryogenic temperature (>60 K) where KQS-based systems degrade significantly due to increased thermal energy.

Cryogenic flexibility addresses the inherent temperature limitations of KQS. Triazolyl-isophthalate MOFs ([Cu_2_(Et-trz-ia)_2_], [Cu_2_(^n^ Pr-trz-ia)_2_]) exhibit a temperature-responsive pore gating effect. At 77 K, selectivity declines from 4 to 5 (at *T*_exp_ = 30 K) to 1.6–2.4, but uptake increases from 0.2 to 1.2 mmol g^–1^, their performance levels consistently exceed commercial cryogenic distillation (S_D2/H2_ = 1.4 at 24 K).

These mechanisms are complementary rather than competing, suggesting that future designs should integrate multiple strategies. Key design criteria are (1) select linker size to achieve 3.0–4.5 Å pores for strong KQS; (2) prioritize Ni^2+^ over Co^2+^ for CAQS, since the higher partial positive charge leads to higher polarization and stronger adsorbate–adsorbent interactions; (3) validate CAQS materials through explicit metal–adsorbate interaction modeling rather than IAST, which fails for strong binding sites. This study clarifies how structural properties impact separation performance and provides guidance for developing MOFs suited to specific operational performance such as maximum selectivity, extended temperature windows, or enhanced throughput.

## Supplementary Material



## Notes

Experimental information, characterization data from ligand synthesis, structures and crystallographic details, PXRD patterns, XPS, gas adsorption data, TDS, SEM, EDAX, and ICP results are provided as PDF (PDF)
