# Supplementary material for: Influence of Structural Determinants on Dihydrogen Adsorption and Isotopologue Separation in Nanoporous Metal–Organic Frameworks
Source: ACS Appl Mater Interfaces. 2026 Feb 25;18(11):16854–62. doi: 10.1021/acsami.5c24726 (PMC13022808; doi:10.1021/acsami.5c24726)
Supplement: Supplementary file 1 [file am5c24726_si_001.pdf]

Supplementary Information

Influence of Structural Determinants on Dihydrogen Adsorption and Isotopologue Separation in Nanoporous Metal-Organic Frameworks

Sibo Chetry[a], *Prantik Sarkar*[b,c], *Mahnaz Bakhtian*[a], *Michael Hirscher*[b,d], *Harald Krautscheid*[a*]

[a] Faculty of Chemistry, Leipzig University, Johannisallee 29, 04103 Leipzig, Germany

[b] Max-Planck-Institute for Intelligent Systems, Heisenbergstrasse 3, D-70569 Stuttgart, Germany

[c] Institute of Separation Science and Technology, Friedrich-Alexander-Universität Erlangen-Nürnberg (FAU), Erlangen 91058, Germany

[d] Advanced Institute for Materials Research (WPI-AIMR), Tohoku University, Aoba-ku, Sendai, 980-8577, Japan

*Email: krautscheid@rz.uni-leipzig.de

Table of Contents

S1. Chemicals 3

S2. Ligand Synthesis 4

S3. Structural Details Based on Single Crystal Structure Data 5

S3.1. Calculation of Open Metal Sites (OMS) density 9

S4. Powder X-ray Diffraction 10

S5. SEM, EDAX and ICP-OES 12

S6. X-Ray Photo Electron Spectroscopy 15

S7. Gas Sorption Isotherms and Isosteric Heat of Adsorption 16

S8. Thermal Desorption Spectroscopy (TDS) 22

S9. IAST Calculation 29

S10. References 31

S1. Chemicals

All chemicals were commercially available.

Chemical	Formula	Vendor	Purity
Methanol	CH_3OH	VWR HPLC Grade	99.99%
Diethyl ether	$C_4H_{10}O$	VWR HPLC Grade	99.99%
Ammonia (30% aqueous solution)	NH_3	Acros Organics	99.95%
Silver oxide	Ag_2O	Acros Organics	99.95%
Copper acetate	$Cu(CH_3COO)$	Acros Organics	≥98%
Copper chloride. dihydrate	$CuCl_2 \cdot 2H_2O$	TCI	≥98%
Cobalt acetate tetrahydrate	$Co(CH_3COO)_2 \cdot 4H_2O$	Sigma Aldrich	≥98%
Nickel nitrate hexahydrate	$Ni(NO_3)_2 \cdot 6H_2O$	Sigma Aldrich	≥98%
Cobalt nitrate hexahydrate	$Co(NO_3)_2 \cdot 6H_2O$	Sigma Aldrich	≥98%
Iodine	I_2	TCI	99.99%
Toluene	C_7H_8	VWR HPLC Grade	99.99%
Mesitylene	C_9H_{12}	Acros Organics	98%
Acetonitrile	CH_3CN	VWR	≥99%
Acetylacetone	$C_5H_8O_2$	Acros Organics	>97%
Sodium hydride	NaH	TCI	>98%
Citric acid	$C_6H_8O_7$	Sigma Aldrich	≥99%
Potassium hydroxide pellets	KOH	Sigma Aldrich	≥90%
Sodium azide	NaN_3	Acros Organics	≥99.5%
Zinc bromide	$ZnBr_2$	Sigma Aldrich	≥99.99 %
Hydrazine monohydrate	$N_2H_4 \cdot H_2O$	Acros Organics	100 % (64 % Hydrazine)
2,5-Dioxido-1,4-benzenedicarboxylate	$C_8H_6O_6$	BLD Pharma	98 %

S2. Ligand Synthesis

Citric acid and 2,5-dioxido-1,4-benzenedicarboxylate were obtained commercially.

The synthesis of protonated triazolyl isophthalate and benzoate linkers followed established methods.[1-8] The same procedure can be applied to substituted triazolyl-benzoate linkers by replacing amino-isophthalic acid with amino-benzoic acid.[1,5]

The tetramethyl bipyrazolate linker was synthesized according to procedures reported in the literature.[6-8]

For the methyl tetrazolate linker, a water-based method for preparing 5-substituted 1H-tetrazoles from nitriles, introduced by Demko and Sharpless, was employed.[9]

S3. Structural Details Based on Single Crystal Structure Data

The crystal structure data of UTSA-16(Co),[12] Ni-MOF-74(Co)[13], Ag-Bpz[6], Cu- Tetrazolate,[14] $[Cu_2(^{n}Pr\text{-trz-ia})_2]$, $([Cu_2(Et\text{-trz-ia})_2])$,[2] and Cu-4py-Me[15] were all retrieved from the CCDC database. Visualization of the MOF structures was performed using Diamond software.[10]

All pore diameters (taking into account the van der Waals radii) and the presence of open metal sites (OMS) were calculated using the Zeo++ high throughout python-based package.[11]

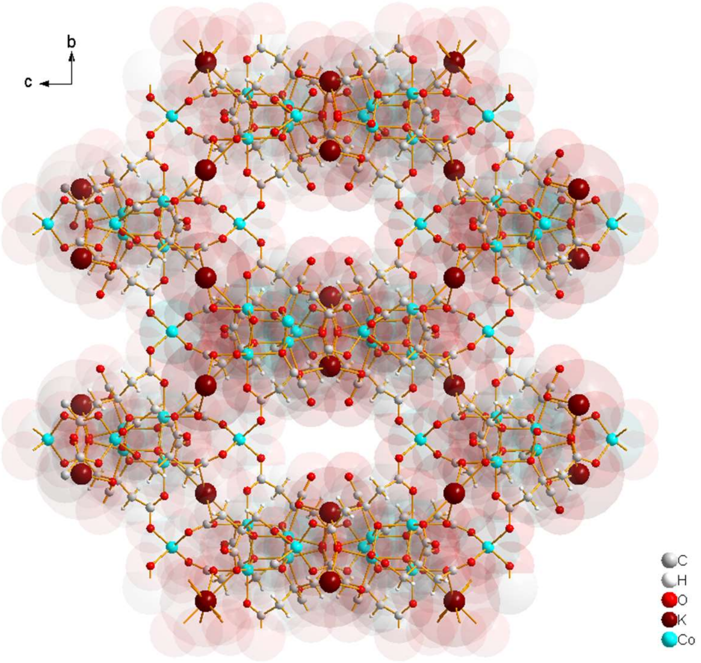

Figure S1. Ball-and-stick model of UTSA-16(Co) based on single crystal data.[12] The model with view along the crystallographic *a* direction shows how the framework creates a network of connected pores and uses cobalt ions as coordination centers for citrate linkers. Co - cyan, K - dark red, O - red, C - gray, H - white.

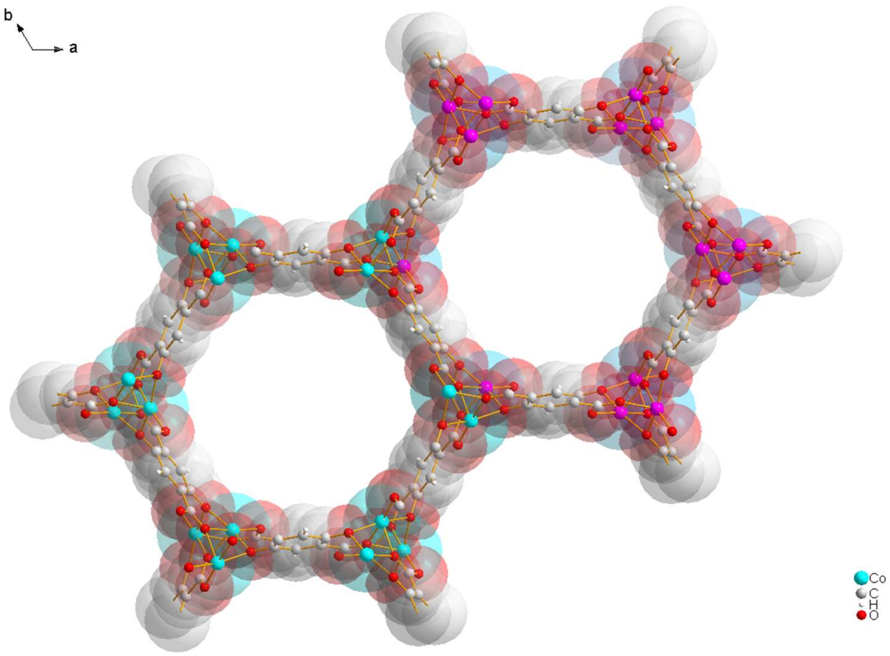

Figure S2. Ball-and-stick model of MOF-74(Co) based on single crystal data (some of the Co atoms were substituted with Ni purely for illustrative purposes. There is no actual single crystal data of the bimetallic Ni-MOF-74(Co); it serves only as a conceptual visualization).[13] Co - cyan, Ni - magenta, O - red, C - gray, H - white.

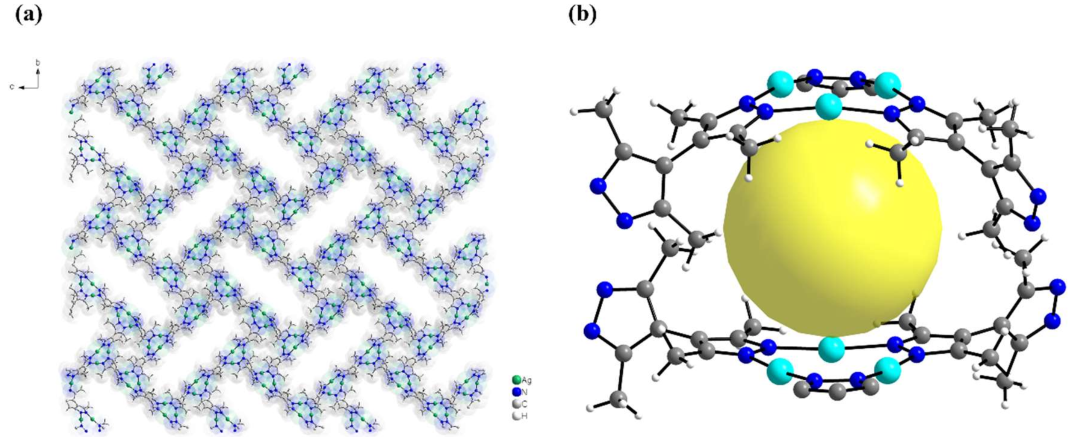

Figure S3. (a) Ball-and-stick model of Ag-Bpz based on single crystal data,[6] with two views. Left: General view of framework structure with Ag -turquoise, C -gray, N - blue, H - white. **(b)** Magnified view where the void is shown with a yellow sphere symbolizing the size of the pore.

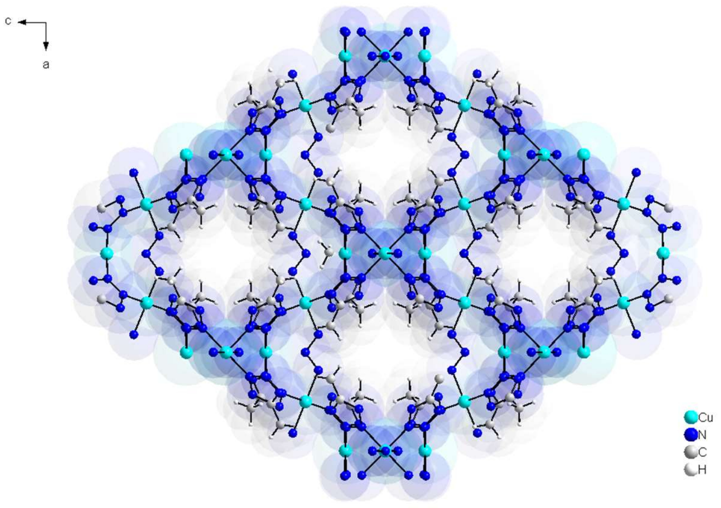

Figure S4. Ball-and-stick model of Cu-tetrazolate based on single crystal data.[14]
Cu - cyan, N - blue, C - gray, H - white.

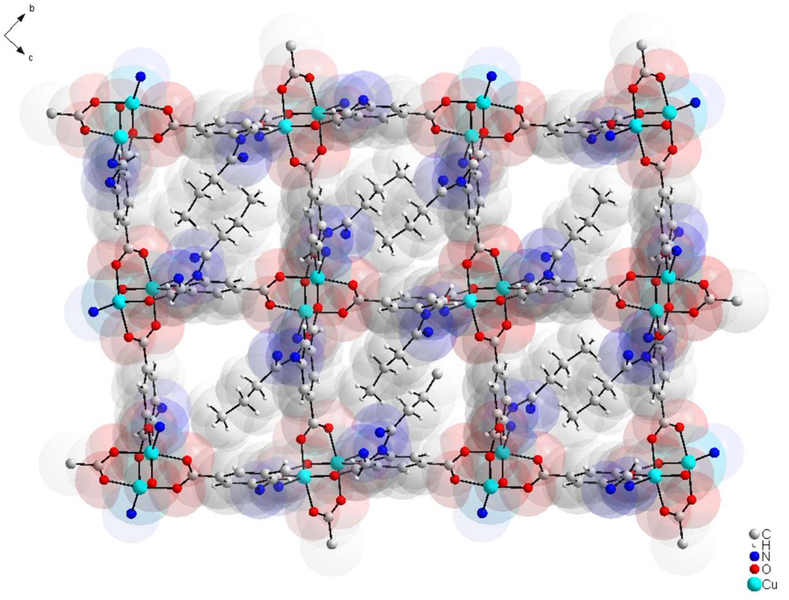

Figure S5. Ball-and-stick model of [Cu_2(^{n}Pr-trz-ia)$_2$] based on single crystal data,[2] view along the crystallographic *a* direction. Cu - turquoise, O - red, N - blue, C - gray, H - white.

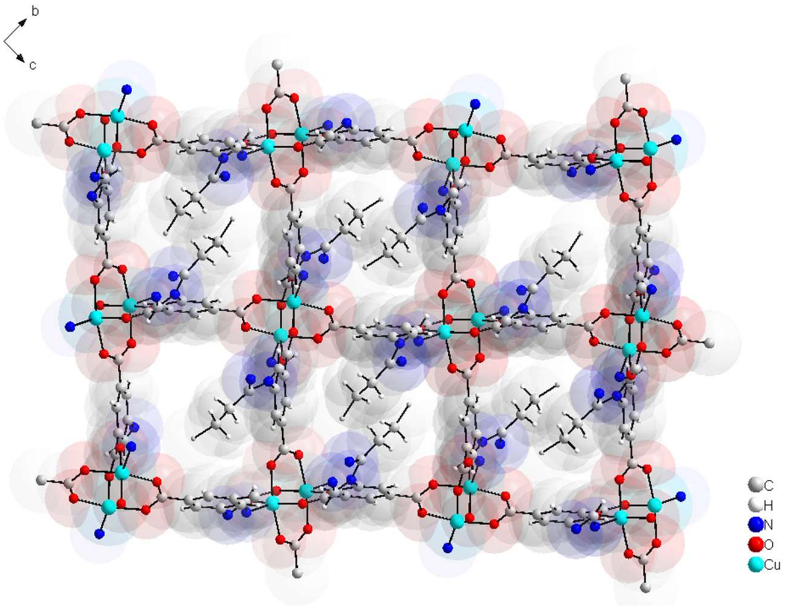

Figure S6. Ball-and-stick model of [Cu_2(Et-trz-ia)$_2$] MOF based on single crystal data.[2] Cu - turquoise, O - red, N - blue, C - gray, H - white.

There is no single crystal structure data for [Cu_2(Et-trz-ia)$_2$]. Therefore, the structural model was derived based on the structure of [Cu_2(^{n}Pr-trz-ia)$_2$],[2] with the n-propyl groups replaced by ethyl groups for the structure diagram. The unit cell dimensions, determined by X-ray powder diffraction, were used in the model.

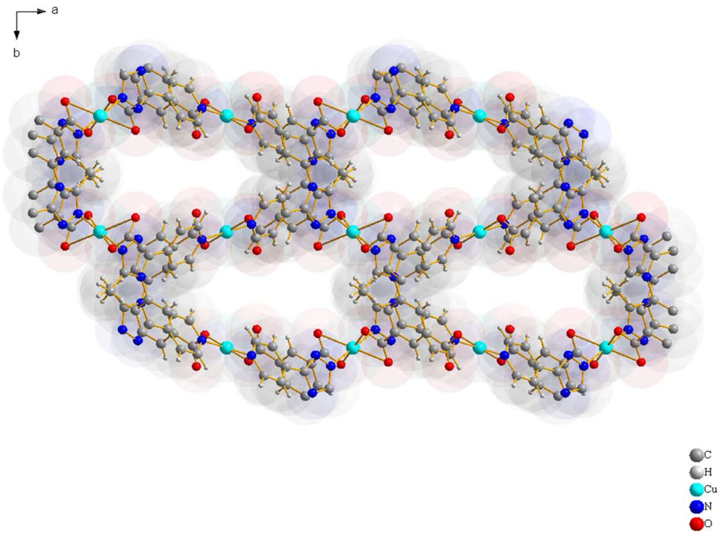

Figure S7. Ball-and-stick model of [Cu-4Py-Me] based on single crystal data.[15]
Cu - turquoise, O - red, N - blue, C - gray, H - white.

S3.1. Calculation of Open Metal Sites (OMS) density

The density of OMS is calculated by the following equation.

$OMS\ density = Z{\cdot}m\ /\ \mathrm{N_A}{\cdot}V$

where Z is the number of formula units in the unit cell, m is the number of metal atoms per formula unit, $\mathrm{N_A}$ is Avogadro's number and V is the unit cell volume.

Table S1. Crystallographic details of various MOFs

MOF	UTSA-16(Co)	Ni-MOF-74(Co)	Ag-Bpz	Cu-Tetrazolate	[Cu_2(Et-trz-ia)$_2$]	[Cu_2(^{n}Pr-trz-ia)$_2$]	Cu-4py-Me
Space Group	$I\bar{4}2d$	$R3$	$I\bar{4}3d$	$Fd\bar{3}m$	$P2_1/c$	$P2_1/c$	$P2_1/c$
Crystal System	tetra-gonal	trigonal	cubic	cubic	mono-clinic	mono-clinic	mono-clinic
Sum Formula	$C_{12}H_8Co_3KO_{20}$	$C_8H_2Co_2O_6$	$C_{10}H_{12}Ag_2N_4$	$C_4H_6CuN_8$	$C_{24}H_{18}Cu_2N_6O_8$	$C_{26}H_{22}Cu_2N_6O_8$	$C_{16}H_{10}CuN_4O_4$
Formula Weight (g mol^{-1})	688.08	311.96	403.96	229.69	645.52	673.58	385.82
a in pm	1306.91(4)	2613.2	2723.3(2)	18.961(7)	1089(1)	1087.8(2)	1400.9(1)
b in pm					1199(1)	1257.9(2)	1331.91(8)
c in pm	3015.7(1)	672.2			1459(1)	1411.4(2)	1483.0(1)
β (°)					109.9(1)	110.02(2)	93.116(7)
V in 10^6 pm^3	5150.9(3)	3975.3	20196(2)	2627.6(13)	1793.27(3)	1814.5(5)	2762.9(4)
Calc. Density (g cm^{-3})	1.774	1.177	1.594	1.742	1.196	1.233	0.927
Z	8	9	48	1	2	2	4
Pore channel (Å)	3.4-4.5	11	3.4-8.0	3.0-3.5	3.4	3.4	5.5
Open Metal Sites Density	-	7.5 mmol cm^{-3} *	7.9 mmol cm^{-3}	-	-	-	-

*: Calculated for pure MOF-74(Co)

S4. Powder X-ray Diffraction

PXRD patterns were obtained at r.t. using a STADI-P diffractometer (STOE & Cie. GmbH) with Cu-$K_{\alpha 1}$ radiation (λ = 1.54060 Å). Glass capillaries (Hilgenberg, outer diameters 0.5 to 0.7 mm) were used to prepare samples for these studies.

PXRD analysis was conducted specifically for Ag-Bpz, $[Cu_2(^{n}Pr\text{-}trz\text{-}ia)_2]$ and $[Cu_2(Et\text{-}trz\text{-}ia)_2]$ due to the flexibility of these MOFs. Structural changes, if any, were recorded both before and after the adsorption experiment.

The Pawley refinement was carried out using TOPAS (Bruker AXS).[16] All the peaks are matched with the simulated ones which indicate all MOF are phase pure.

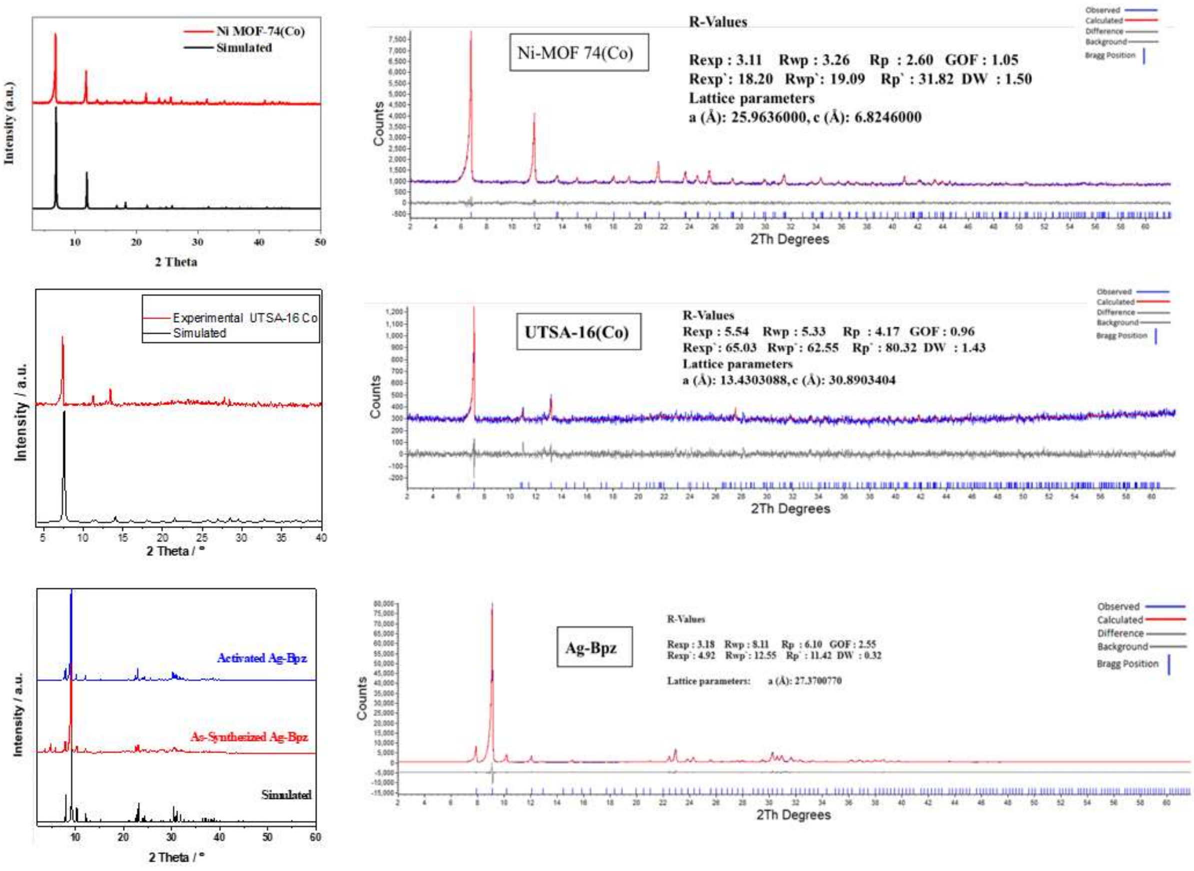

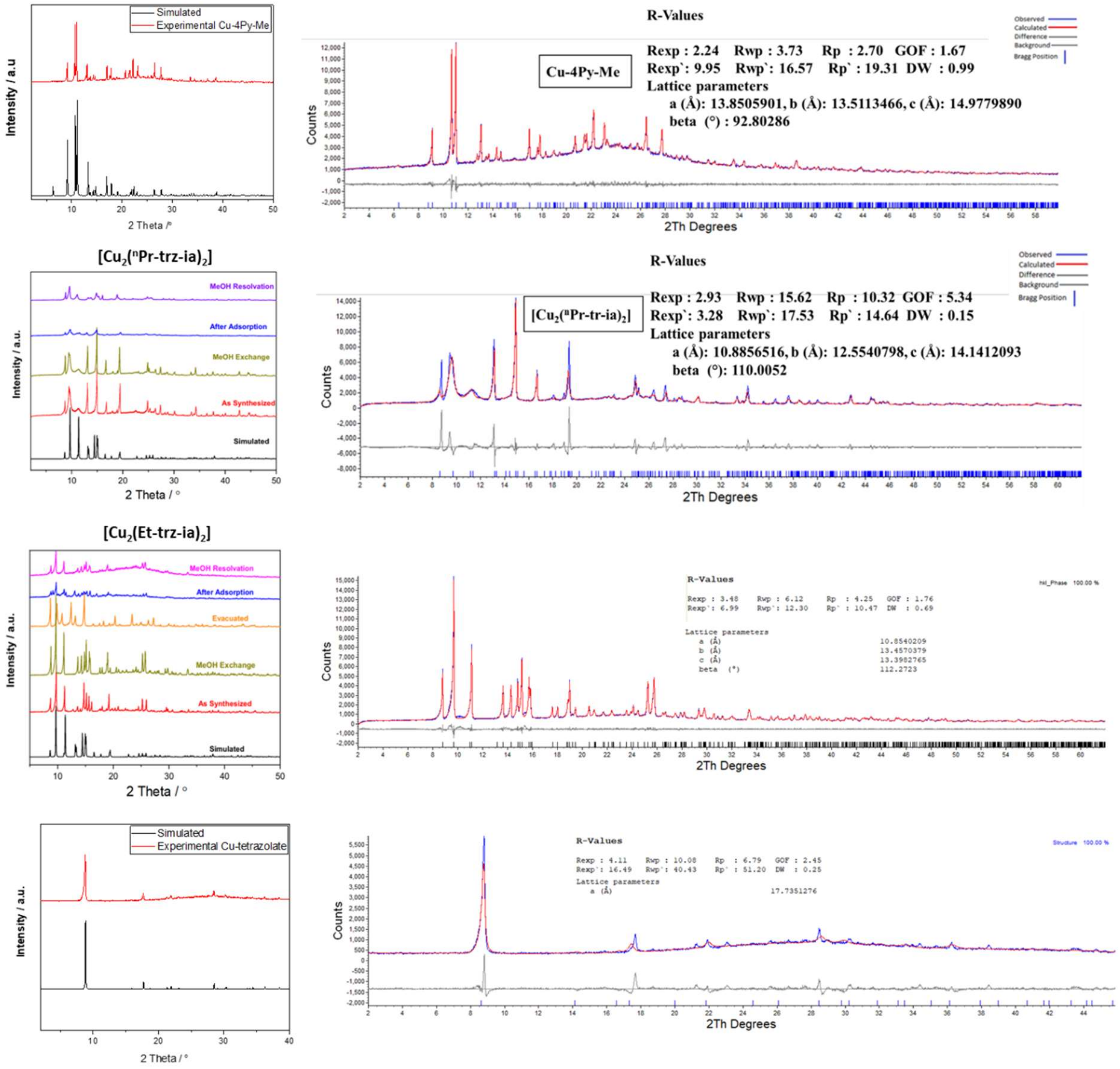

Figure S8. PXRD patterns of all MOFs included in this study in comparison with simulated patterns based on single crystal data. Also presented are the results of the Pawley refinements.

S5. SEM, EDAX and ICP-OES

SEM images were captured using a Phenom Pharos G2 Desktop FEG-SEM Tabletop field emission gun scanning electron microscope equipped with a back scattered electron detector. Acceleration voltage 15 kV.

In addition to SEM-EDAX measurements, the nickel and cobalt concentrations in Ni-MOF-74(Co) were also analyzed quantitatively using ICP-OES on a PerkinElmer Optima 8000 instrument. For ICP-OES sample preparation, the MOFs were digested in nitric acid. The obtained Co and Ni percentages confirm a Ni:Co molar ratio of 1:1: Co 19.1 wt-% (calc. for $NiCoC_8H_2O_6$ 18.9 wt-%); Ni 18.4 wt-% (calc. 18.8 wt-%).

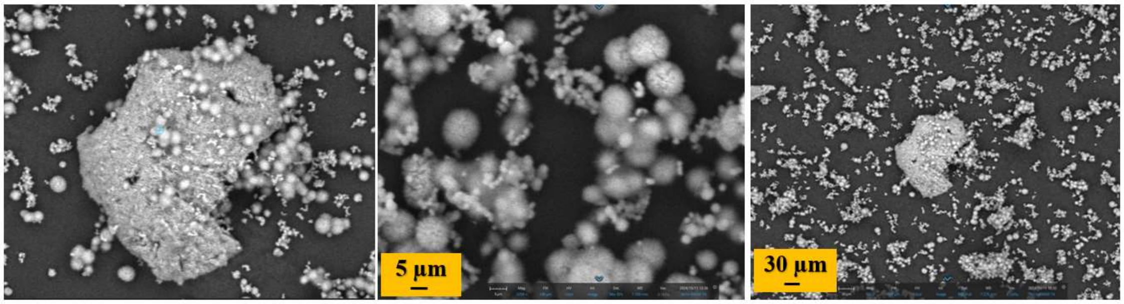

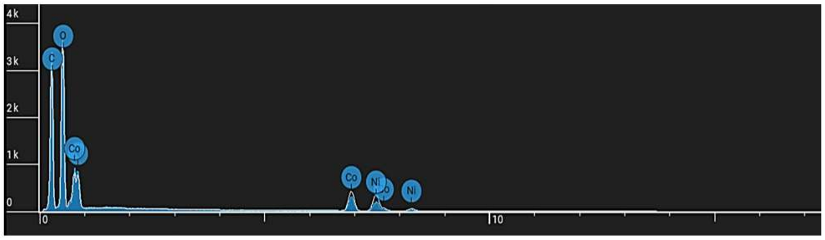

Element	Weight%
Ni	18.14
Co	19.24
O	32.87
C	29.76

Figure S9. SEM image and EDAX analysis of Ni-MOF-74(Co).

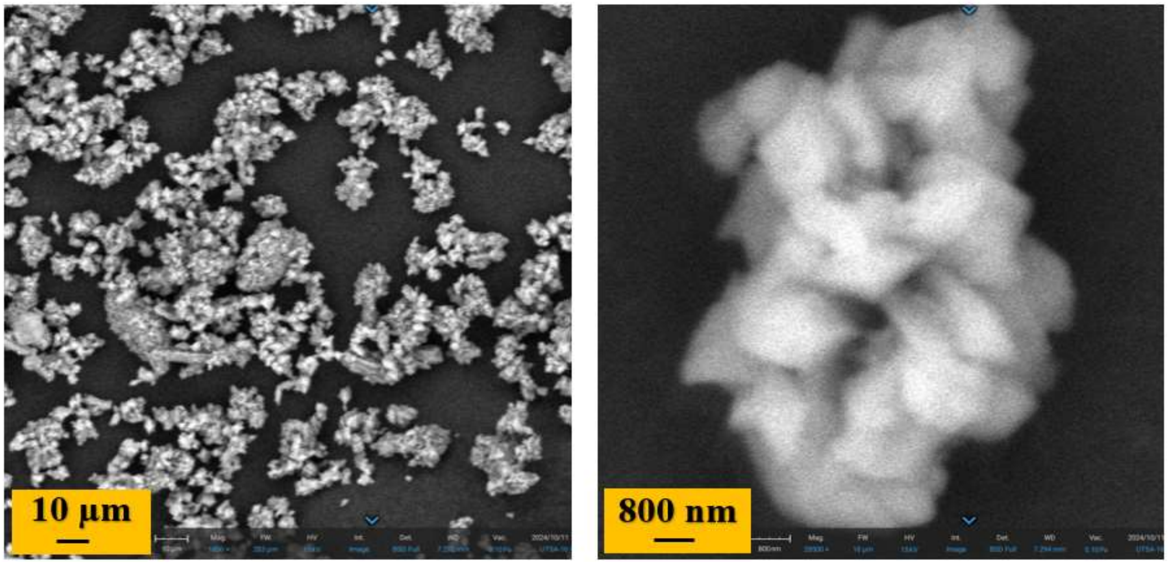

Figure S10. SEM image analysis of UTSA-16(Co).

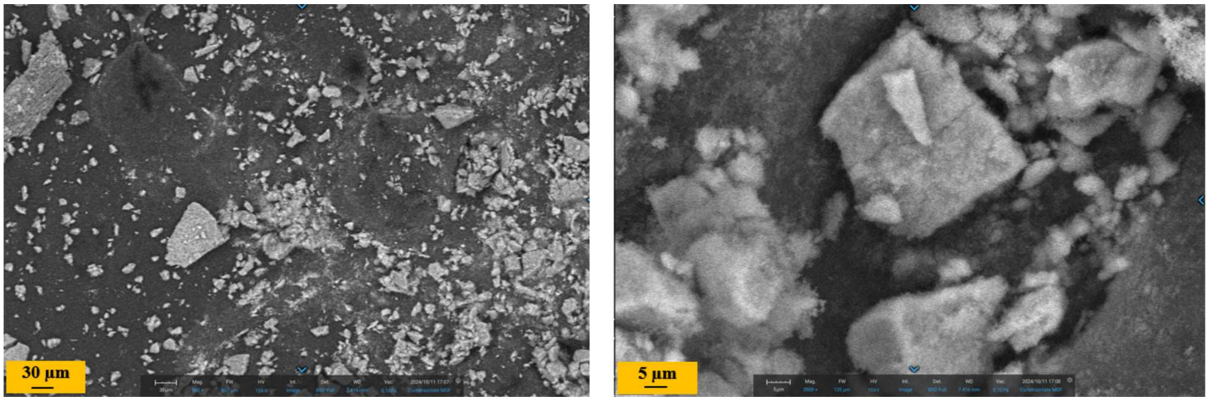

Figure S11. SEM image of Cu-tetrazolate.

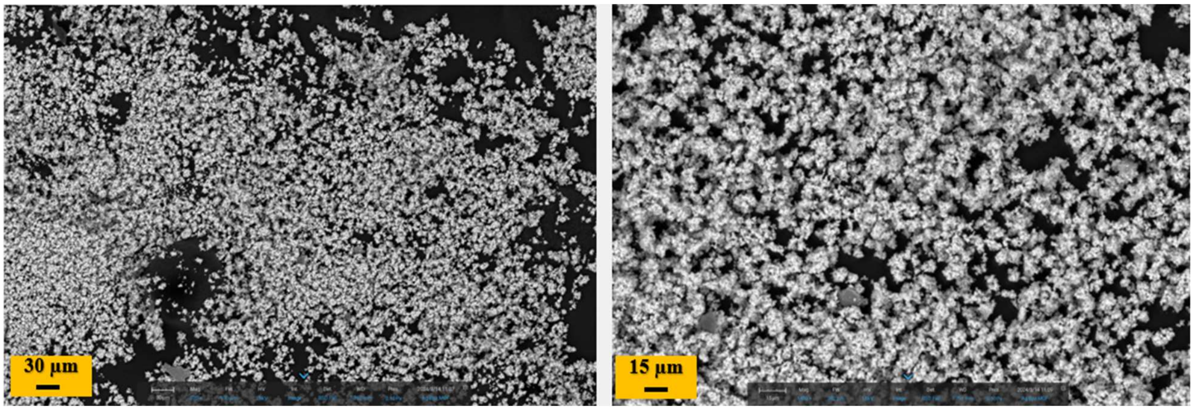

Figure S12. SEM image of Ag-Bpz

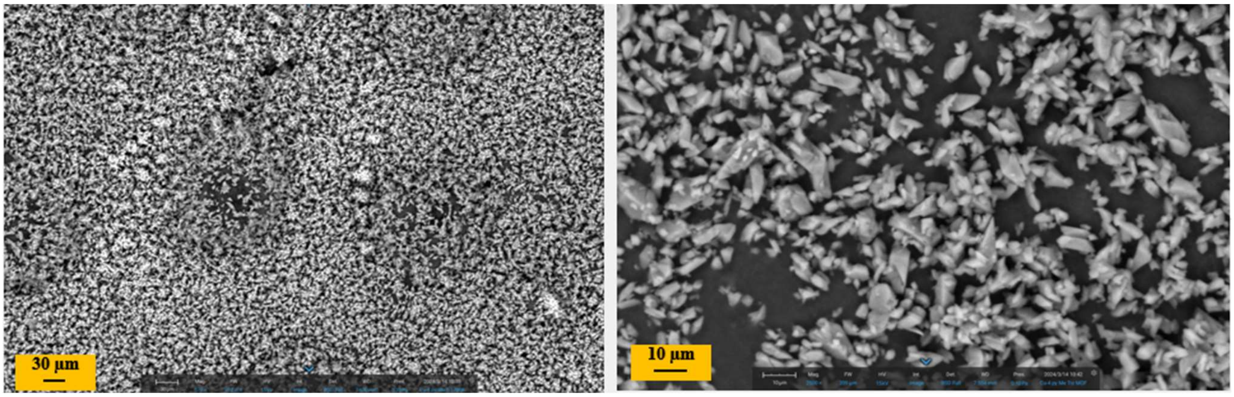

Figure S13. SEM image of Cu-4Py-Me.

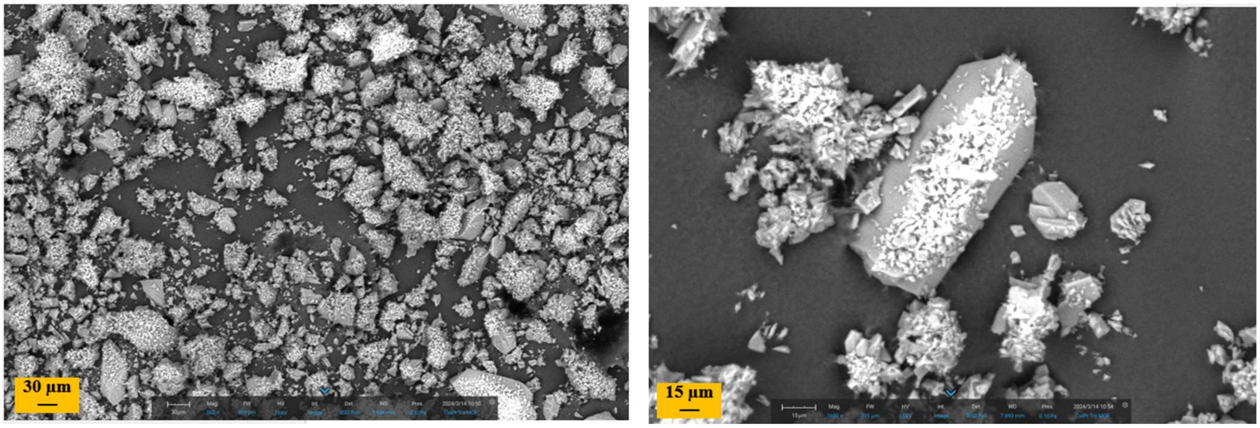

Figure S14. SEM image of [Cu$_2$(^{n}Pr-trz-ia)$_2$].

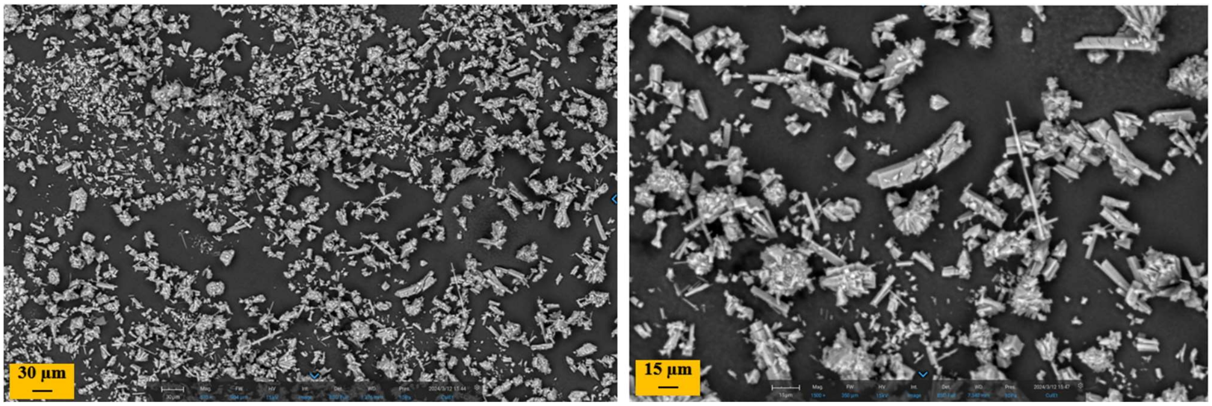

Figure S15. SEM image of [Cu$_2$(Et-trz-ia)$_2$].

S6. X-Ray Photo Electron Spectroscopy

X-ray photoelectron spectroscopy (XPS) analysis was conducted using a K Alpha+ XPS system (Thermo Fisher Scientific Instruments, UK). For the measurements, monochromatic Al-Kα radiation was utilized, generated in a sealed X-ray tube with a beam current of 6 mA and an acceleration voltage of 12 kV. Instrument calibration was verified using the Ag 3d peak at 352 eV. The spot size for analysis on the sample was 400 µm. Binding energies were referenced to the C1s peak at 284.8 eV.

The Ag $3d_{5/2}$ peak is observed around 367.6 eV, which is typical for silver in the oxidation state +I. The corresponding Ag $3d_{3/2}$ peak appears around 373.7 eV, confirming the presence of Ag^{I}.[17]

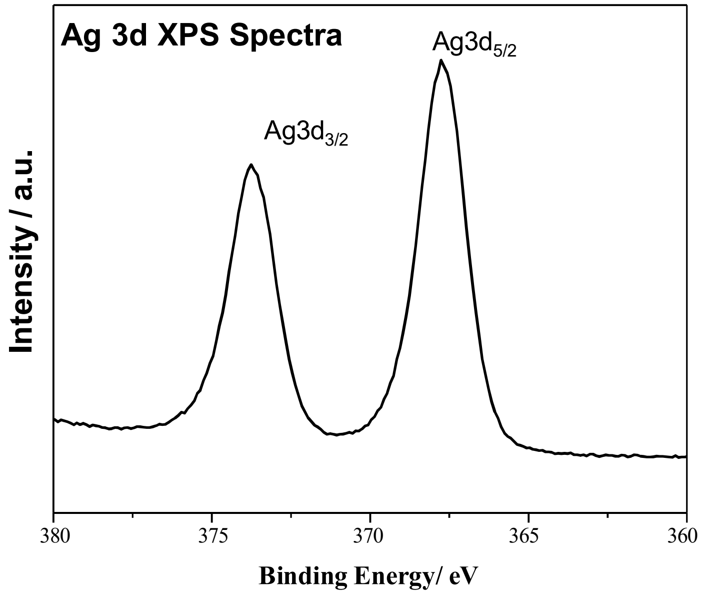

Figure S16. XPS spectrum of Ag-Bpz showing the characteristic binding energies for the Ag^{I} 3d core.

S7. Gas Sorption Isotherms and Isosteric Heat of Adsorption

Before adsorption measurements, the samples (50-100 mg) were degassed in two-stage dynamic vacuum, using a rotary pump at 10^{-2} mbar, followed by a turbo molecular pump (TMP) at 10^{-5} mbar. $[Cu_2(Et\text{-}trz\text{-}ia)_2]$, UTSA-16 (Co) and $[Cu_2(^{n}Pr\text{-}trz\text{-}ia)_2]$ were degassed at room temperature for 24 h after solvent exchange with MeOH. Ag-Bpz, Cu-tetrazolate and Cu-4Py-Me were activated at 373 K for 24 h in dynamic vacuum. Ni-MOF-74(Co) was activated at 423 K for 24 h.

The N_2 sorption measurements were conducted in the relative pressure range $10^{-5} \leq p/p_0 \leq 1$ at 77 K (liquid nitrogen). Data evaluation was performed using BEL Master 6.3.0.0 software for BELSORP max G instrument. H_2 (99.995%), D_2 (99.98%), N_2 gas (99.998%) was used for physisorption and He gas of high purity (99.998%) used to determine the dead volume after the measurement.

High resolution H_2 low-pressure isotherms up to 100 kPa were measured at 77 and 97 K using a 3P cryoTune. CO_2 sorption was measured at 195 K using a dry ice bath in EtOH.

Heat of adsorption was calculated using Clausius-Clapeyron equation, details of the methodology are published elsewhere.[18]

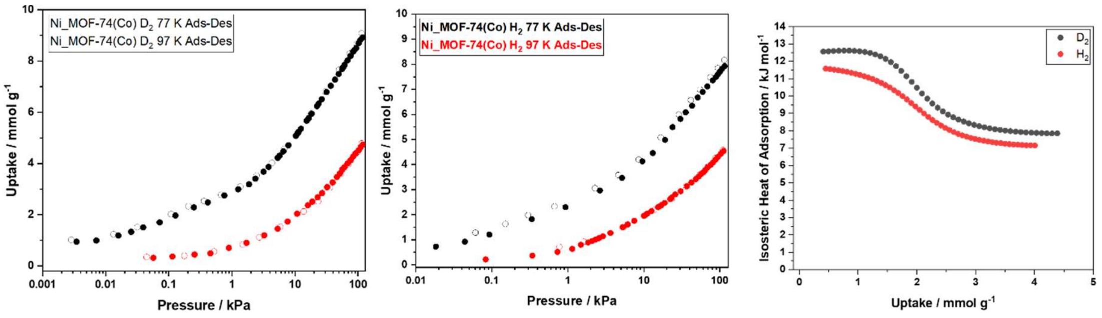

Figure S17. Sorption isotherms of Ni-MOF-74(Co); left: D_2 sorption isotherms recorded at 77 K and 97 K; middle: H_2 sorption isotherms recorded at 77 K and 97 K, closed symbols – adsorption, open symbol – desorption; right: isosteric heat of adsorption for D_2 and H_2.

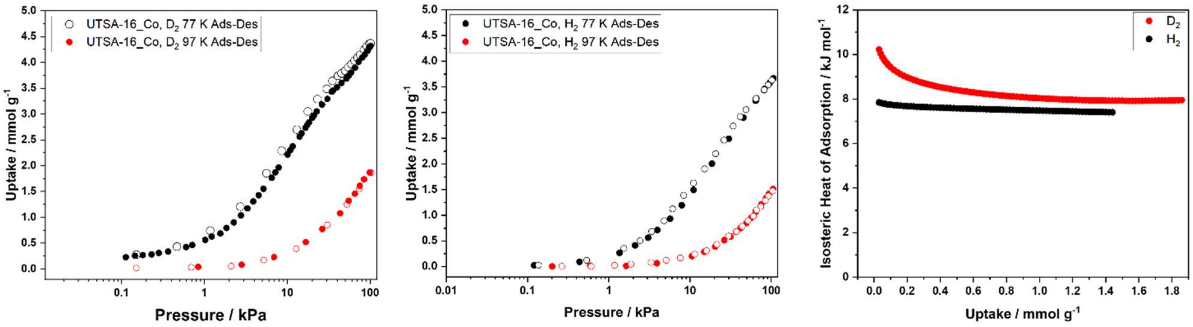

Figure S18. Sorption isotherms of UTSA-16(Co); left: D_2 sorption isotherms recorded at 77 K and 97 K; middle: H_2 sorption isotherms recorded at 77 K and 97 K, closed symbols – adsorption, open symbol – desorption; right: isosteric heat of adsorption for D_2 and H_2.

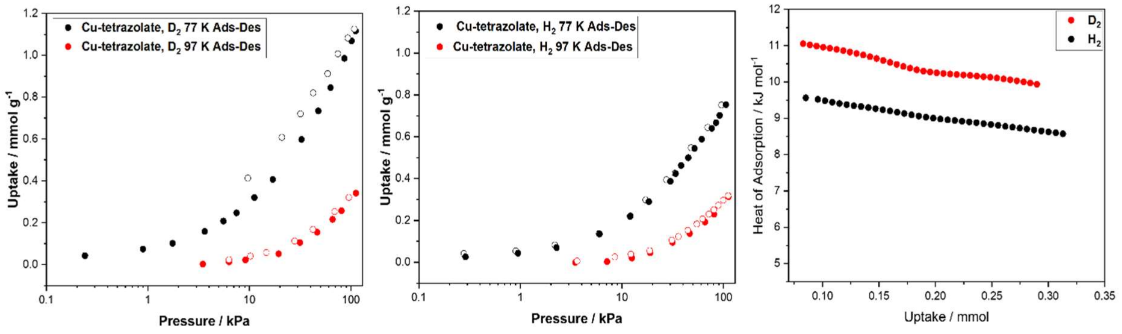

Figure S19. Sorption isotherms of Cu-tetrazolate; left: D_2 sorption isotherms recorded at 77 K and 97 K; middle: H_2 sorption isotherms recorded at 77 K and 97 K, closed symbols – adsorption, open symbol – desorption; right: isosteric heat of adsorption for D_2 and H_2.

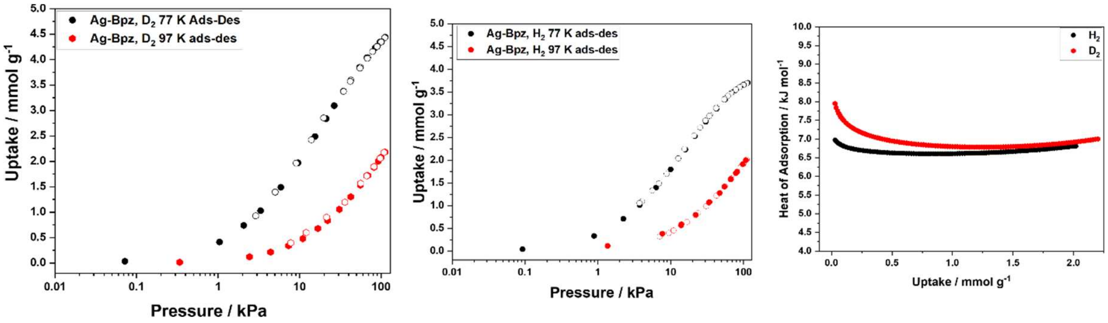

Figure S20. Sorption isotherms of Ag-Bpz; left: D_2 sorption isotherms recorded at 77 K and 97 K; middle: H_2 sorption isotherms recorded at 77 K and 97 K, closed symbols – adsorption, open symbol – desorption; right: isosteric heat of adsorption for D_2 and H_2.

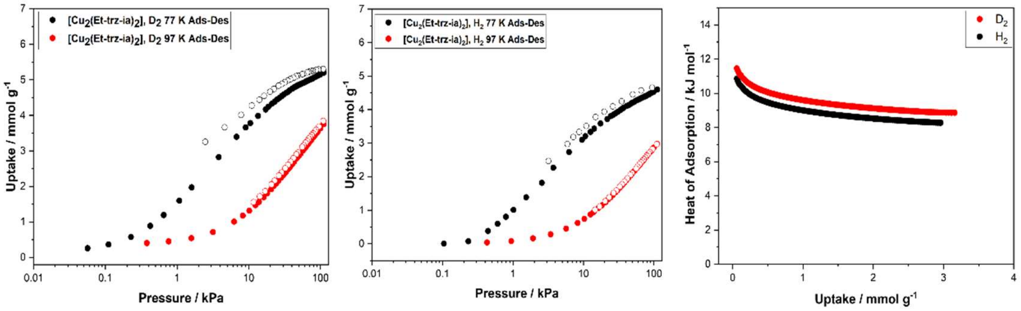

Figure S21. Sorption isotherms of $[Cu_2(Et\text{-}trz\text{-}ia)_2]$; left: D_2 sorption isotherms recorded at 77 K and 97 K; middle: H_2 sorption isotherms recorded at 77 K and 97 K, closed symbols – adsorption, open symbol – desorption; right: isosteric heat of adsorption for D_2 and H_2.

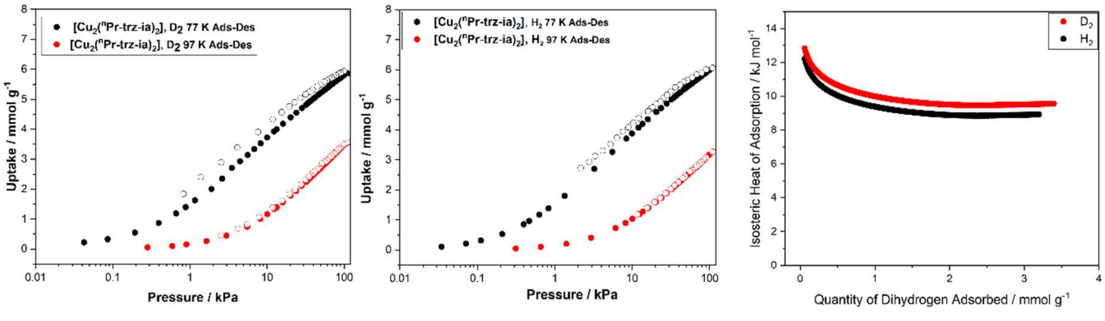

Figure S22. Sorption isotherms of $[Cu_2(^{n}Pr\text{-}trz\text{-}ia)_2]$; left: D_2 sorption isotherms recorded at 77 K and 97 K; middle: H_2 sorption isotherms recorded at 77 K and 97 K, closed symbols – adsorption, open symbol – desorption; right: isosteric heat of adsorption for D_2 and H_2.

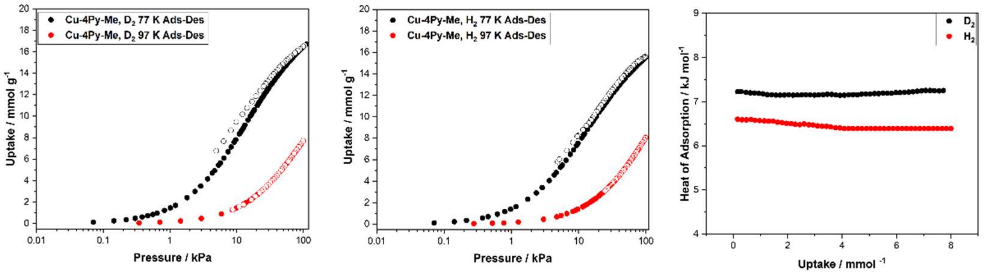

Figure S23. Sorption isotherms of Cu-4Py-Me; left: D_2 sorption isotherms recorded at 77 K and 97 K; middle: H_2 sorption isotherms recorded at 77 K and 97 K, closed symbols – adsorption, open symbol – desorption; right: isosteric heat of adsorption for D_2 and H_2.

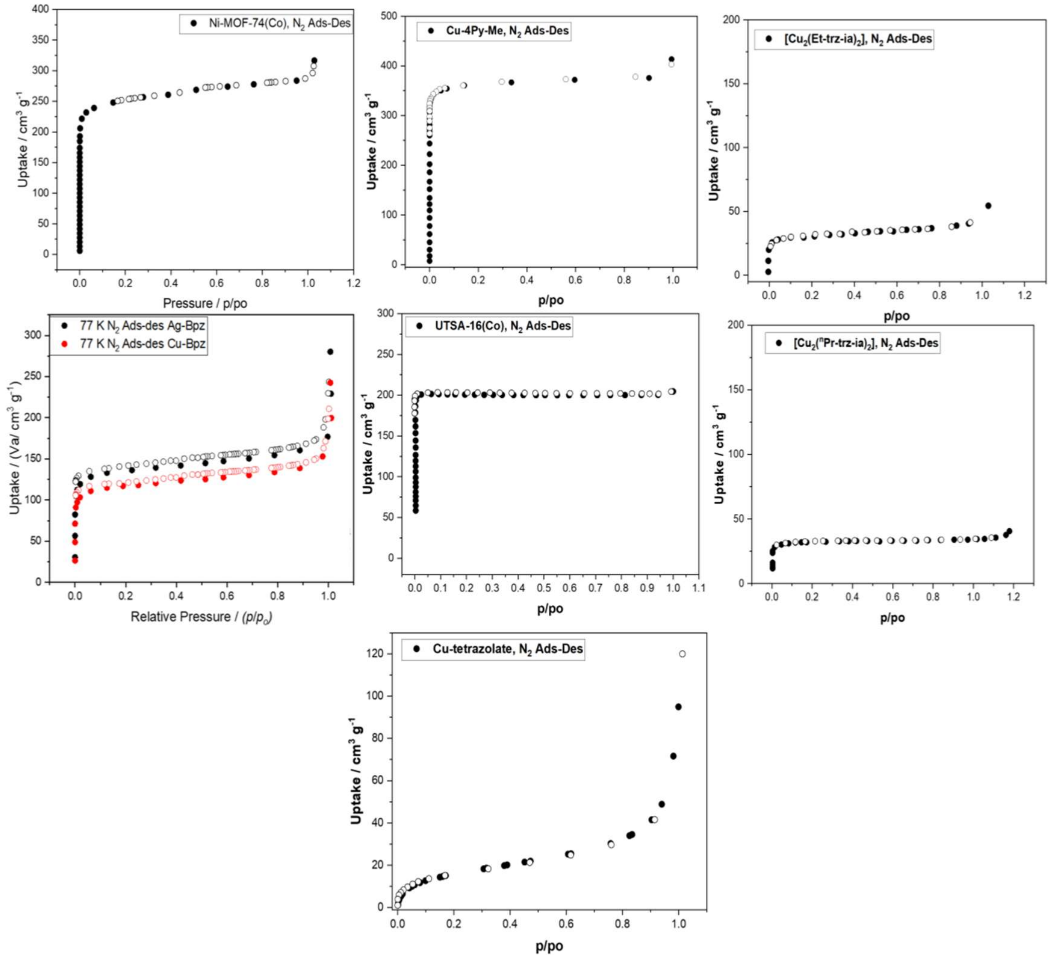

Figure S24. N_2 sorption isotherms measured at 77 K.

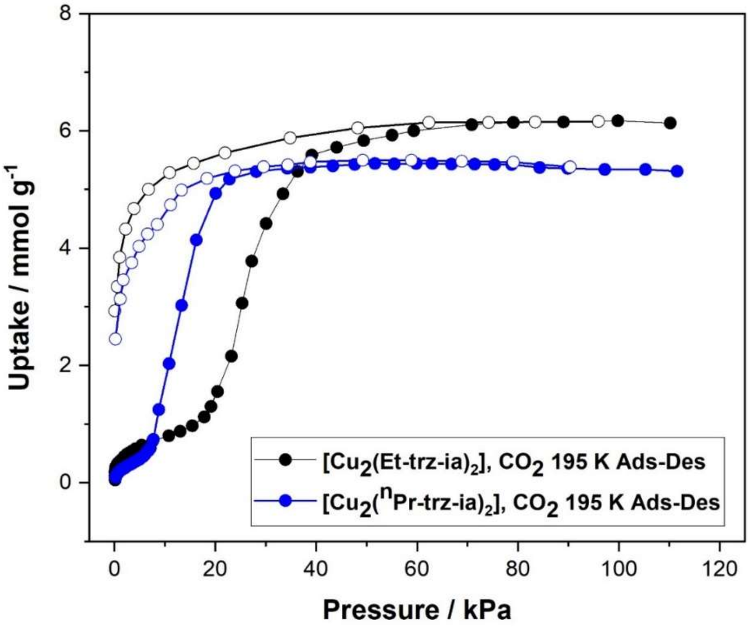

Figure S25. CO_2 sorption isotherms measured at 195 K and up to 100 kPa for [Cu_2(^{n}Pr-trz-ia)$_2$] and [Cu_2(Et-trz-ia)$_2$].

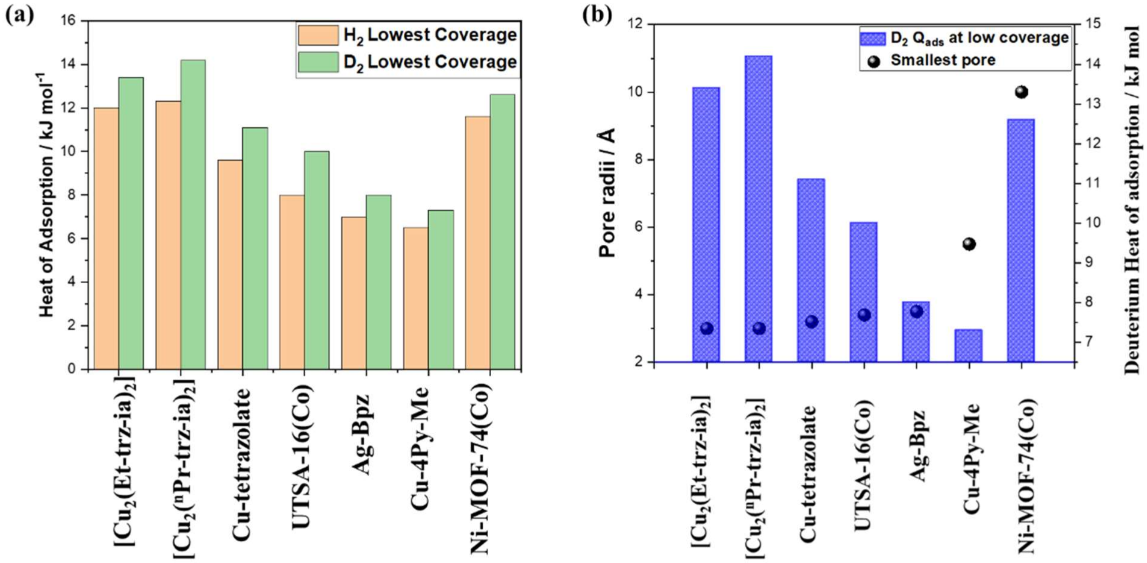

Figure S26. (a) Isosteric heat of adsorption (Q_{ads}) for H_2 and D_2, determined from adsorption measurements at 77 K and 97 K at the lowest coverage, (b) information about the pore diameter (smaller pore size in case of bimodal pores) and D_2 heat of adsorption at the lowest coverage.

Table S2. Summary of Adsorption Properties of the MOFs included in this study.

MOFs	Uptake at 77 K and 100 kPa (in mmol g^{-1})		Calculated Heat of Adsorption Q_{ads} (in kJ mol^{-1}) at low coverage	
	H_2	D_2	H_2	D_2
UTSA-16(Co)	3.7	4.4	8	10
Cu-4Py-Me	15.6	16.5	6.5	7.3
Ni-MOF-74(Co)	7.4	8.2	11.6	12.6
Cu-tetrazolate	0.7	1.2	9.6	11.1
[Cu_2(Et-trz-ia)$_2$]	4.9	5.1	12.0	13.4
[Cu_2(^{n}Pr-trz-ia)$_2$]	5.9	6.1	12.3	14.2
Ag-Bpz	3.7	4.5	7.0	8.0

S8. Thermal Desorption Spectroscopy (TDS)

For TDS experiments, an in-house Cryogenic Thermal Desorption Spectroscopy system was utilized.[19] Approximately 2-4 mg of the sample was taken and activated overnight under ultra-high vacuum conditions (10^{-5} mbar). Subsequently, the sample was exposed to pure D_2, H_2, and 1:1 D_2/H_2 mixtures at specific exposure temperatures (T_{exp}), pressures (p_{exp}) and exposure times (t_{exp}). Following gas exposure, non-adsorbed gases were removed using a turbomolecular pump, and the sample was cooled to 20 K to preserve the adsorbed state. The final step involves applying a linear heating ramp (6 K·min^{-1}) and monitoring the desorbing gas continuously by a quadrupole mass spectrometer. The quantity of desorbed gas is directly proportional to the area under the desorption peak, which can be quantitatively calculated after calibration of the instrument with a $Pd_{95}Ce_5$ alloy.[19]

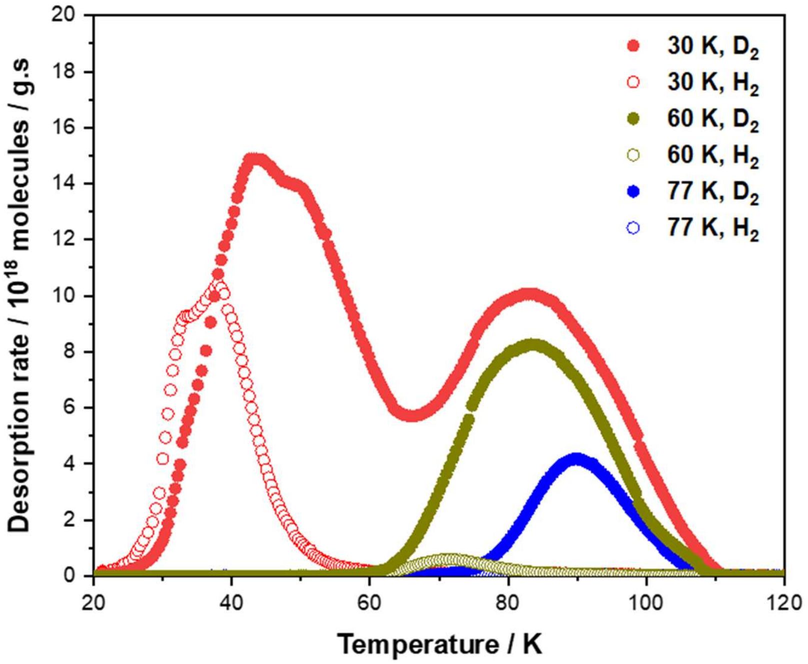

Figure S27. TDS analysis of Ni-MOF-74(Co) showing the desorption rate as a function of temperature, including varying exposure temperatures T_{exp} (30 K, 60 K, and 77 K) and gases (H_2 and D_2) at p_{exp} = 10 mbar for t_{exp} = 10 minutes. Closed symbols for D_2, open symbols for H_2.

.

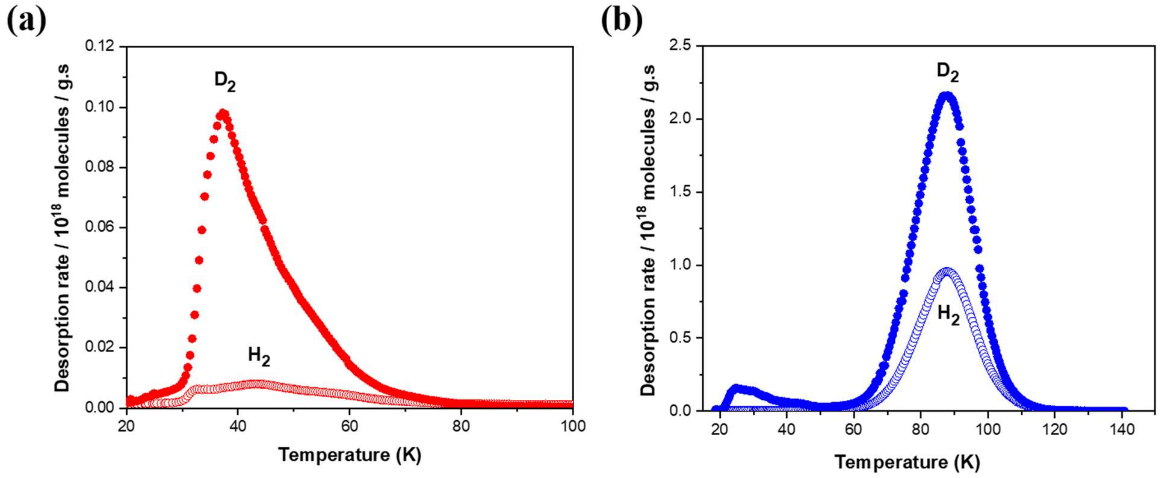

Figure S28. TDS analysis of $[Cu_2(Et\text{-}trz\text{-}ia)_2]$ showing the desorption rate as a function of temperature for various conditions, including different exposure temperatures (**a**) T_{exp} = 30 K, (**b**) T_{exp} = 77 K, and gases (H_2 and D_2) at p_{exp} = 10 mbar for t_{exp} = 10 minutes. Closed symbols for D_2 and open symbols for H_2.

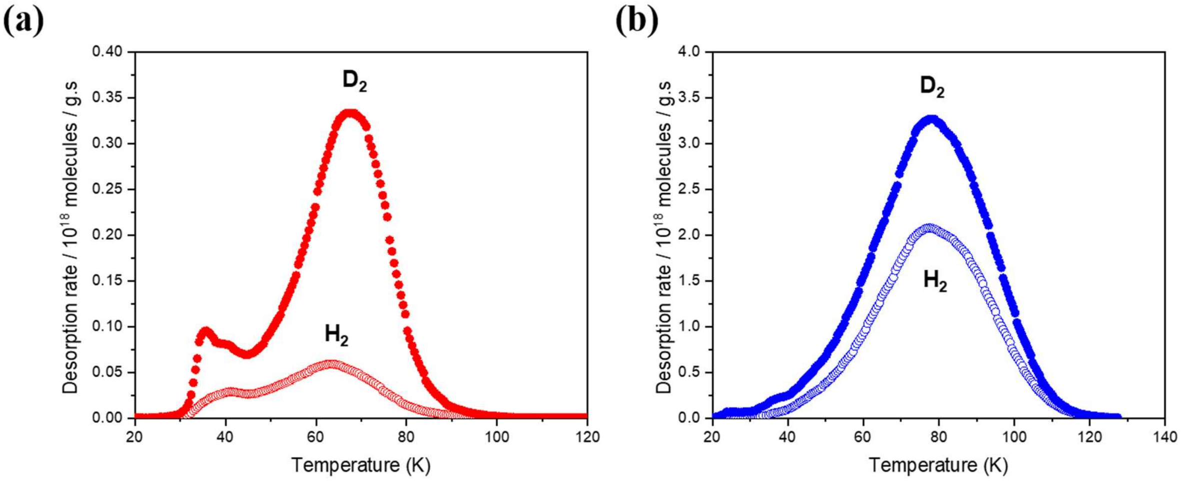

Figure S29. TDS analysis of $[Cu_2(^{n}Pr\text{-}trz\text{-}ia)_2]$ showing the desorption rate as a function of temperature for various conditions, including different exposure temperatures (**a**) T_{exp} = 30 K, (**b**) T_{exp} = 77 K, and gases (H_2 and D_2) at p_{exp} = 10 mbar for t_{exp} = 10 minutes. Closed symbols for D_2 and open symbols for H_2.

.

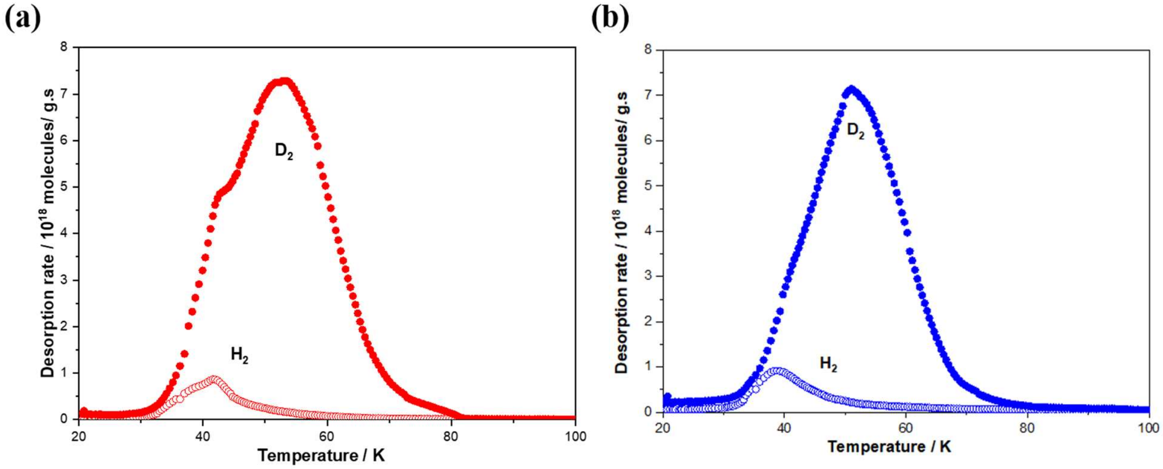

Figure S30. TDS analysis of UTSA-16(Co) showing the desorption rate as a function of temperature for various conditions, including different exposure temperatures (**a**) T_{exp} = 30 K, (**b**) T_{exp} = 77 K, and gases (H_2 and D_2) at p_{exp} = 10 mbar for t_{exp} = 10 minutes. Closed symbols for D_2 and open symbols for H_2.

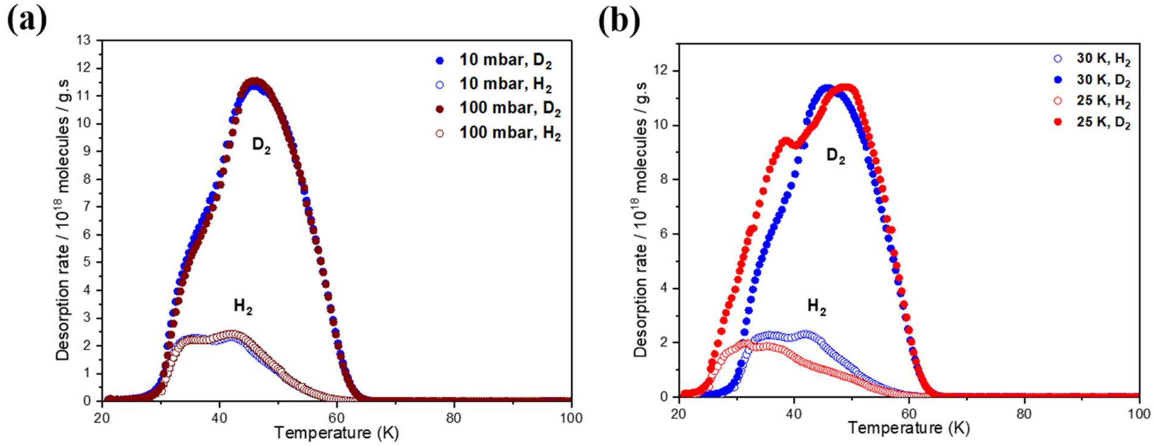

Figure S31. TDS analysis of Ag-Bpz showing the desorption rate as a function of temperature for t_{exp} = 10 minutes and different gases (H_2 and D_2) (**a**) at T_{exp} = 30 K including different pressures (p_{exp} = 10 mbar and 100 mbar) (**b**) at p_{exp} = 100 mbar including different temperatures (T_{exp} = 25 K and 30 K). Closed symbols for D_2 and open symbols for H_2.

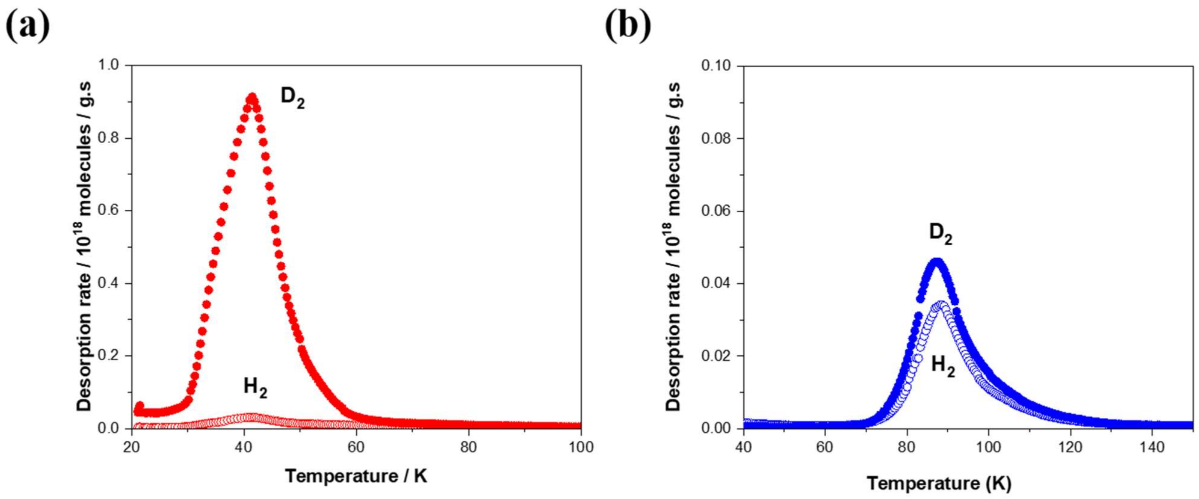

Figure S32. TDS analysis of Cu-tetrazolate showing the desorption rate as a function of temperature for various conditions, including different temperatures (**a**) T_{exp} =30 K, (**b**) T_{exp} =77 K, and gases (H_2 and D_2) at p_{exp} = 100 mbar for t_{exp} = 10 minutes. Closed symbols for D_2 and open symbols for H_2.

Table S3 summarizes the exposure conditions, including T_{exp}, t_{exp}, and p_{exp}, used during dosing with a 1:1 D_2/ H_2 gas mixture, together with the corresponding TDS results in terms of H_2 uptake, D_2 uptake, total uptake, and selectivity. In a typical TDS experiment, the sample is first exposed to the isotopologue mixture at the defined T_{exp}, t_{exp}, and p_{exp}. Prior to initiating the desorption measurement, the chamber is briefly evacuated, usually at the exposure temperature, to remove non adsorbed gas, as the mass spectrometer operates only under high vacuum conditions. Owing to the configuration of the vacuum system, exposure pressures are limited to values below atmospheric pressure. Consequently, the applied exposure conditions cannot be directly compared with adsorption isotherms measured under equivalent conditions. Exposure is typically carried out at pressures of 10 or 100 mbar with loading times ranging from 10 to 60 min. When the total uptake remains unchanged with increasing pressure and exposure time, equilibrium loading is achieved, as observed for UTSA-16(Co). In some cases, the total uptake stabilizes while the isotope selectivity continues to increase with exposure time, as seen for Ag-Bpz, indicating that equilibration of D_2 and H_2 occupancy at the adsorption sites requires longer times. This behavior reflects an isotopic exchange process following initial adsorption, which proceeds until equilibrium dictated by the isotopic difference in adsorption enthalpy is reached. For flexible frameworks, exposure at temperatures above the framework opening temperature leads to substantially enhanced gas uptake, as exemplified by [Cu_2(^{n}Pr/Et-trz-ia)$_2$].

Table S3. Summarized results of the H_2 and D_2 uptake values and selectivities for all the MOFs measured at varying exposure temperatures T_{exp} (25-77 K), exposure pressures p_{exp} (10-100 mbar) and exposure times t_{exp} (10-60 min).

Sample	T_{exp} (K)	t_{exp} (min)	p_{exp} (mbar)	H_2 uptake (mmol g^{-1})	D_2 uptake (mmol g^{-1})	Selectivity $S_{D2/H2}$	Total Uptake (mmol g^{-1})
UTSA-16 Co	30	10	10	0.18	2.51	13.28	2.70
	30	10	100	0.18	2.71	14.84	2.92
	30	60	100	0.20	2.63	13.02	2.84
	77	10	100	0.24	2.50	10.12	2.75
[Cu-4py-Me]	30	10	100	1.19	9.67	8.10	10.86
Ni MOF-74(Co)	30	10	10	2.40	10.59	4.39	12.99
	60	10	10	0.15	3.14	20.66	3.29
	77	10	10	0.02	1.12	52.40	1.14
Cu-Tetrazolate	30	10	100	0.012	0.20	16.23	0.22
	77	10	100	0.01	0.016	1.16	0.03
$[Cu_2(Et\text{-}trz\text{-}ia)_2]$	23	10	100	0.01	0.05	5.4	0.06
	30	10	100	0.00	0.02	5.66	0.03
	77	10	100	0.32	0.77	2.38	1.09
$[Cu_2(^nPr\text{-}trz\text{-}ia)_2]$	30	10	100	0.02	0.10	4.55	0.12
	77	10	100	1.21	1.95	1.61	3.16
Ag-Bpz	25	10	10	0.63	4.65	7.37	5.28
	30	10	10	0.71	3.89	5.44	4.60
	30	10	100	0.72	3.80	5.26	4.52
	30	30	100	0.52	4.02	7.64	4.54
	30	60	100	0.49	3.78	7.60	4.28

Table S4. Comparison of D_2/H_2 selectivity $S_{D2/H2}$ and D_2 uptake of several porous materials.

MOFs	p_{exp} [mbar]	T_{exp} [K]	$S_{D2/H2}$ 1:1 Mixture	D_2 Uptake [mmol g^{-1}]	Ref.
MFU-4	2	60	7.5	-	20
Ag^{I}-Zeolite-Y	10	90	10	3.0	21
MOF-74(Ni)	10	30	6	14.5	22
	10	77	19	4.0	22
MOF-74-IM-10	10	30	6.5	12	22
	10	77	26	2.8	22
Co(pyz)[M(CN)$_4$], M=Pd, Ni, Pt	10	25	21.7 (Pd), 17.8 (Ni), 16.1 (Pt)	10.5 (Pd), 11.2 (Ni), 9.7 (Pt)	23
MOF-74(Co)	30	30	3.7	10	24
	80	10	6.3	1.0	24
Defect-HKUST-1	25	20	26	18	25
CoFA	1000	25	26	7.0	26
MIL-53(Al)	10	25	2.83	22.1	27
DUT-8(Ni)	800	23.3	11.6	9.4	28
Py@COF-1	26	22	9.7	0.5	29
MOF-303	1000	25	21	18.6	30
oIFP-3	10	30	2.41	1.6	31
IFP-1	10	30	1.69	4.3	31
Cocryst1	10	30	7.7	4.72	32
CC3	10	30	1.7	3.6	32
6FT-RCC3	10	30	2.2	2.8	32
6ET-RCC3	10	30	3.9	0.3	32
SIFSIX-3-Zn<br>SIFSIX-3-Ni	25<br>25	20<br>20	53.8<br>1.9	0.9<br>0.8	33
STAM-1	26	20	9.9	2.8	33
KAUST-7	10	20	9.8	0.3	33
Ni-MOF-74(Co)	10	30	4.4	10.5	This work
	10	77	52	1.12	
UTSA-16(Co)	100	30	14.8		This work

Cu-4Py-Me	100	30	8	9.7	This work
Cu-tetrazolate	100	30	16.2	0.2	This work
$[Cu_2(Et\text{-}trz\text{-}ia)_2]$	100	30	5.66	0.02	This work
	100	77	2.38	0.77	
$[Cu_2(^nPr\text{-}trz\text{-}ia)_2]$	100	30	4.55	0.10	This work
	100	77	1.61	1.95	
Ag-Bpz	100	30	5.26	3.80	This work

S9. IAST Calculation

Ideal Adsorbed Solution Theory (IAST) is a widely used thermodynamic framework for predicting the adsorption behavior of gas mixtures based solely on pure-component isotherms. It assumes that the adsorbed phase behaves like an ideal solution and enables estimation of multicomponent adsorption equilibria without requiring mixture adsorption data.[34,35] IAST is particularly valuable for evaluating separation performance in porous materials such as MOFs. In this study, IAST calculations were carried out using the open-source Python package pyGAPS,[34] which provides a flexible platform for isotherm modeling and mixture analysis.

Table S5 compares the selectivity ($S_{D2/H2}$) derived from IAST calculation to the TDS derived selectivity. The observed divergence between IAST-predicted selectivities and experimental TDS results highlights the distinct physical regimes probed by each method, particularly within OMS-containing frameworks. IAST assumes thermodynamic equilibrium and an ideal mixing of adsorbates on a homogeneous surface; however, the presence of OMS introduces significant energetic heterogeneity and strong specific interactions that deviate from ideal solution behavior, often leading IAST to underestimate the competitive advantage of the heavier isotopologue at low coverage. Consequently, for systems relying on strong chemical affinity (CAQS), TDS selectivities offer a more rigorous representation of the achievable separation performance under realistic, dynamic operating conditions than equilibrium-based IAST predictions.

Table S5. Summarized IAST results of the H_2 and D_2 uptake values and selectivities $S_{D2/H2}$ for the investigated MOFs calculated based on pure component adsorption isotherms at 77 K with assumption of a 1:1 H_2:D_2 equimolar mixture and 100 mbar pressure. For comparison, the selectivities and uptake values experimentally determined by TDS are given.

MOFs	calc. IAST Selectivity	calc. IAST D_2 Uptake (mmol/g)	calc. IAST H_2 uptake (mmol/g)	Exp. Selectivity $S_{D2/H2}$ (TDS)	Exp. D_2 uptake (mmol/g) (TDS)	Exp. H_2 uptake (mmol/g) (TDS)
UTSA-16 (Co)	3.7	1.7	0.34	10.12	2.50	0.24
Cu-4Py-Me	1.0	7.3	0.29	-	-	-
Ni MOF-74 (Co)	2.6*	3.5	1.1	52.40*	1.12*	0.02
Cu-tetrazolate	1.4	0.19	0.15	1.16	0.016	0.01
[Cu_2(Et-trz-ia)$_2$]	2.0	2.4	1.06	2.38	0.77	0.32
[Cu_2(^{n}Pr-trz-ia)$_2$]	1.2	2.7	1.2	1.61	1.95	1.21
Ag-Bpz	1.1	1.0	0.86	-	-	-

* The difference between the calculated and experimental results for Ni-MOF-74(Co) arises from the fact that the IAST model does not include the effects of open metal sites.

S10. References

(1) Lässig, D.; Lincke, J.; Krautscheid, H. Highly Functionalised 3,4,5-Trisubstituted 1,2,4-Triazoles for Future Use as Ligands in Coordination Polymers. *Tetrahedron Lett.* **2010**, *51* (4), 653–656. https://doi.org/10.1016/j.tetlet.2009.11.098.

(2) Kobalz, M.; Lincke, J.; Kobalz, K.; Erhart, O.; Bergmann, J.; Lässig, D.; Lange, M.; Möllmer, J.; Gläser, R.; Staudt, R.; Krautscheid, H. Paddle Wheel Based Triazolyl Isophthalate MOFs: Impact of Linker Modification on Crystal Structure and Gas Sorption Properties. *Inorg. Chem.* **2016**, *55* (6), 3030–3039. https://doi.org/10.1021/acs.inorgchem.5b02921.

(3) Lincke, J.; Lässig, D.; Kobalz, M.; Bergmann, J.; Handke, M.; Möllmer, J.; Lange, M.; Roth, C.; Möller, A.; Staudt, R.; Krautscheid, H. An Isomorphous Series of Cubic, Copper-Based Triazolyl Isophthalate MOFs: Linker Substitution and Adsorption Properties. *Inorg Chem.* **2012**, *51* (14), 7579–7586. https://doi.org/10.1021/ic3003228.

(4) Ainsworth, C.; Hackler, R. E. Alkyl-1,3,4-Oxadiazoles. *J. Org. Chem.* **1966**, *31* (10), 3442–3444. https://doi.org/10.1021/jo01348a531.

(5) Lincke, J.; Lässig, D.; Moellmer, J.; Reichenbach, C.; Puls, A.; Moeller, A.; Gläser, R.; Kalies, G.; Staudt, R.; Krautscheid, H. A Novel Copper-Based MOF Material: Synthesis, Characterization and Adsorption Studies. *Micropor. Mesopor. Mater.* **2011**, *142* (1), 62–69. https://doi.org/10.1016/j.micromeso.2010.11.017.

(6) Zhang, J. P.; Kitagawa, S. Supramolecular Isomerism, Framework Flexibility, Unsaturated Metal Center, and Porous Property of Ag(I)/Cu(I) 3,3′,5,5′-Tetrametyl-4,4′-Bipyrazolate. *J. Am. Chem. Soc.* **2008**, *130* (3), 907–917. https://doi.org/10.1021/ja075408b.

(7) Boldog, I.; Rusanov, E. B.; Chernega, A. N.; Sieler, J.; Domasevitch, K. V. One- and Two-Dimensional Coordination Polymers of 3,3′,5,5′-Tetramethyl-4,4′-Bipyrazolyl, a New Perspective Crystal Engineering Module. *Polyhedron,* **2001**, *20*, 887–897. https://doi.org/10.1016/S0277-5387(01)00726-4.

(8) Boldog, I.; Rusanov, E. B.; Chernega, A. N.; Sieler, J.; Domasevitch, K. V. Coordination Polymers of Co^{II} and 3,3′,5,5′-Tetramethyl-4,4′-Bipyrazolyl: A Novel Metal–Organic Three-Dimensional Network with Four-Coordinated Planar Vertices. *Dalton Trans.* **2001**, *6*, 893–897. https://doi.org/10.1039/b007183h.

(9) Demko, Z. P.; Sharpless, K. B. *J. Org. Chem.* **2001**, *66*, 7945-7950.

(10) K. Brandenburg; H. Putz. Diamond Version 3.2k; Bonn, **2014**.

(11) Willems, T. F.; Rycroft, C. H.; Kazi, M.; Meza, J. C.; Haranczyk, M. Algorithms and Tools for High-Throughput Geometry-Based Analysis of Crystalline Porous Materials. *Micropor. Mesopor. Mater.* **2012**, *149* (1), 134–141. https://doi.org/10.1016/j.micromeso.2011.08.020.

(12) Xiang, S.; He, Y.; Zhang, Z.; Wu, H.; Zhou, W.; Krishna, R.; Chen, B. Microporous Metal-Organic Framework with Potential for Carbon Dioxide Capture at Ambient conditions. *Nat. commun.*, **2012**, *3*, 954. DOI: 10.1038/ncomms1956.

(13) Pato-Doldán, B.; Rosnes, M. H.; Dietzel, P. D. C. An In-Depth Structural Study of the Carbon Dioxide Adsorption Process in the Porous Metal–Organic Frameworks CPO-27-M. *ChemSusChem.,* **2017**,*10*(8), 1710–1719. https://doi.org/10.1002/cssc.201601752.

(14) Cho, S.; Kim, Y.; Lee, S.; Cho, H.; Park, J.; Hwan Hong, D.; Kwon, K.; Yoo, H.; Choe, W.; Ri Moon, H. Tetrazole-Based Energetic Metal-Organic Frameworks: Impacts of Metals and Ligands on Explosive Properties. *Eur. J. Inorg. Chem.,* **2022**, e202100757. https://doi.org/10.1002/ejic.202100757.

(15) Lässig, D.; Lincke, J.; Moellmer, J.; Reichenbach, C.; Moeller, A.; Gläser, R.; Kalies, G.; Cychosz, K. A.; Thommes, M.; Staudt, R.; Krautscheid, H. A Microporous Copper Metal-Organic Framework with High H_2 and CO_2 Adsorption Capacity at Ambient Pressure. *Angew. Chem. Int. Ed.,* **2011**, *50* (44), 10344–10348. https://doi.org/10.1002/anie.201102329.

(16) Bruker AXS. TOPAS Version 5, 2014, Karlsruhe.

(17) Roy, K.; Vinod, C. P.; Gopinath, C. S. Design and Performance Aspects of a Custom-Built Ambient Pressure Photoelectron Spectrometer toward Bridging the Pressure Gap: Oxidation of Cu, Ag, and Au Surfaces at 1 Mbar O_2 Pressure. *J. Phys. Chem. C.,* **2013**, *117* (9), 4717–4726. https://doi.org/10.1021/jp312706s.

(18) Nuhnen, A.; Janiak, C. A Practical Guide to Calculate the Isosteric Heat/Enthalpy of Adsorption: Via Adsorption Isotherms in Metal-Organic Frameworks, MOFs. *Dalton Trans.*, **2020**, *49*, 10295–10307. https://doi.org/10.1039/d0dt01784a.

(19) Oh, H.; Hirscher, M. Quantum Sieving for Separation of Hydrogen Isotopes Using MOFs. *Eur. J. Inorg. Chem*. **2016**, 4278–4289. https://doi.org/10.1002/ejic.201600253.

(20) Teufel, J.; Oh, H.; Hirscher, M.; Wahiduzzaman, M.; Zhechkov, L.; Kuc, A.; Heine, T.; Denysenko, D.; Volkmer, D. MFU-4 - A Metal-Organic Framework for Highly Effective H_2/D_2 Separation. *Adv. Mater.* **2013**, *25* (4), 635–639. https://doi.org/10.1002/adma.201203383.

(21) Zhang, L.; Wulf, T.; Baum, F.; Schmidt, W.; Heine, T.; Hirscher, M. Chemical Affinity of Ag-Exchanged Zeolites for Efficient Hydrogen Isotope Separation. *Inorg. Chem*. **2022**, *61* (25), 9413–9420. https://doi.org/10.1021/acs.inorgchem.2c00028.

(22) Kim, J. Y.; Balderas-Xicohténcatl, R.; Zhang, L.; Kang, S. G.; Hirscher, M.; Oh, H.; Moon, H. R. Exploiting Diffusion Barrier and Chemical Affinity of Metal-Organic Frameworks for Efficient Hydrogen Isotope Separation. *J. Am. Chem. Soc*. **2017**, *139* (42), 15135–15141. https://doi.org/10.1021/jacs.7b07925.

(23) Ha, J.; Jung, M.; Park, J.; Oh, H.; Moon, H. R. Thermodynamic Separation of Hydrogen Isotopes Using Hofmann-Type Metal-Organic Frameworks with High-Density Open Metal Sites. *ACS Appl. Mater. Interfaces*, **2022**, *14* (27), 30946–30951. https://doi.org/10.1021/acsami.2c07829.

(24) Oh, H.; Savchenko, I.; Mavrandonakis, A.; Heine, T.; Hirscher, M. Highly Effective Hydrogen Isotope Separation in Nanoporous Metal–Organic Frameworks with Open Metal Sites: Direct Measurement and Theoretical Analysis. *ACS Nano*, **2014**, *8* (1), 761–770. https://doi.org/10.1021/nn405420t.

(25) Hu, X.; Ding, F.; Xiong, R.; An, Y.; Feng, X.; Song, J.; Zhou, L.; Li, P.; Chen, C. Highly Effective H_2/D_2 Separation within the Stable Cu(I)Cu(II)-BTC: The Effect of Cu(I) Structure on Quantum Sieving. *ACS Appl. Mater. Interfaces*, **2023**, *15* (3), 3941–3952. https://doi.org/10.1021/acsami.2c18221.

(26) Muhammad, R.; Jee, S.; Jung, M.; Park, J.; Kang, S. G.; Choi, K. M.; Oh, H. Exploiting the Specific Isotope-Selective Adsorption of Metal–Organic Framework for Hydrogen Isotope Separation. *J. Am. Chem. Soc.* **2021**, *143* (22), 8232–8236. https://doi.org/10.1021/jacs.1c01694.

(27) Kim, J. Y.; Zhang, L.; Balderas-Xicohténcatl, R.; Park, J.; Hirscher, M.; Moon, H. R.; Oh, H. Selective Hydrogen Isotope Separation via Breathing Transition in MIL-53(Al). *J. Am. Chem. Soc.* **2017**, *139* (49), 17743–17746. https://doi.org/10.1021/jacs.7b10323.

(28) Bondorf, L.; Fiorio, J. L.; Bon, V.; Zhang, L.; Maliuta, M.; Ehrling, S.; Senkovska, I.; Evans, J. D.; Joswig, J.-O.; Kaskel, S.; Heine, T.; Hirscher, M. Isotope-Selective Pore Opening in a Flexible Metal-Organic Framework. *Sci. Adv.* **2022**, *8* (15). https://doi.org/10.1126/sciadv.abn7035.

(29) Oh, H.; Kalidindi, S. B.; Um, Y.; Bureekaew, S.; Schmid, R.; Fischer, R. A.; Hirscher, M. A Cryogenically Flexible Covalent Organic Framework for Efficient Hydrogen Isotope Separation by Quantum Sieving. *Angew. Chem. Int. Ed.* **2013**, *52* (50), 13219–13222. https://doi.org/10.1002/anie.201307443.

(30) Kim, H.; Jee, S.; Park, J.; Jung, M.; Muhammad, R.; Choi, K.; Oh, H. High D_2/H_2 Selectivity Performance in MOF-303 under Ambient Pressure for Potential Industrial Applications. *Sep. Purif. Technol.* **2023**, *325*, 124660, DOI: 10.1016/j.seppur.2023.124660.

(31) Mondal, S. S.; Kreuzer, A.; Behrens, K.; Schütz, G.; Holdt, H.-J.; Hirscher, M. Systematic Experimental Study on Quantum Sieving of Hydrogen Isotopes in Metal-Amide-Imidazolate Frameworks with Narrow 1-D Channels. *ChemPhysChem.* **2019**, *20* (10), 1311–1315, https://doi.org/10.1002/cphc.201900183.

(32) Liu, M.; Zhang, L.; Little, M. A.; Kapil, V.; Ceriotti, M.; Yang, S.; Ding, L.; Holden, D. L.; Balderas-Xicohténcatl, R.; He, D.; Clowes, R.; Chong, S. Y.; Schütz, G.; Chen, L.; Hirscher, M.; Cooper, A. I. Barely Porous Organic Cages for Hydrogen Isotope Separation. *Science,* **2019**, *366* (6465), 613–620, https://doi.org/10.1126/science.aax7427.

(33) G. Han, Y. Gong, H. Huang, D. Cao, X. Chen, D. Liu, and C. Zhong, Screening of Metal–Organic Frameworks for Highly Effective Hydrogen Isotope Separation by Quantum Sieving. *ACS Appl. Mater. Interfaces*, **2018**, *10* (38), 32128–32132. https://doi.org/10.1021/acsami.8b10201.

(34) Iacomi, P; Llewellyn, P.L. pyGAPS: A Python-Based Framework for Adsorption Isotherm Processing and Material Characterisation. *Adsorption*, **2019,** *25*, 1533–1542. https://doi.org/10.1007/s10450-019-00168-5.

(35) Myers, A.L.; Prausnitz, J.M., Thermodynamics of mixed-gas adsorption. *AIChE J.*, **1965**, *11*, 121-127. https://doi.org/10.1002/aic.690110125.
